# Contrasting genetic diversity between *Planchonella obovata* sensu lato (Sapotaceae) on old continental and young oceanic island populations in Japan

**DOI:** 10.1371/journal.pone.0273871

**Published:** 2022-09-02

**Authors:** Suzuki Setsuko, Kyoko Sugai, Ichiro Tamaki, Koji Takayama, Hidetoshi Kato

**Affiliations:** 1 Department of Forest Molecular Genetics and Biotechnology, Forestry and Forest Products Research Institute, Forest Research and Management Organization, Tsukuba, Ibaraki, Japan; 2 Institute of Agricultural and Life Sciences, Academic Assembly, Shimane University, Matsue, Shimane, Japan; 3 Gifu Academy of Forest Science and Culture, Mino, Gifu, Japan; 4 Department of Botany, Graduate School of Science, Kyoto University, Sakyo-ku, Kyoto, Japan; 5 Makino Herbarium, Tokyo Metropolitan University, Hachioji, Tokyo, Japan; The University of Auckland - City Campus: University of Auckland, NEW ZEALAND

## Abstract

Genetic diversity of plant populations on islands is likely to be influenced by characteristics such as island origin (oceanic or continental) and their age, size, and distance to continental landmasses. In Japan, *Planchonella obovata* sensu lato which is found on both continental and oceanic islands of varying age, size, and distance to East Asian continental areas—is an ideal system in which to investigate the factors influencing genetic diversity of island plant species. In this study, we examined the genetic diversity of *P*. *obovata s*.*l*. populations, in the context of the species population genetic structure, demography, and between island migration, from 668 individuals, 28 populations and 14 islands including both continental (the Yaeyama Islands) and oceanic islands (the Daito, Bonin, and Volcano Islands) using 11 microsatellite markers. The Yaeyama and Volcano Islands respectively had the highest and lowest genetic diversity, and island origin and age significantly affected genetic diversity. Clustering analysis revealed that populations were grouped into Bonin, Volcano, and Yaeyama + Daito groups. However, Bonin and Volcano groups were distinct despite the relatively short geographical distance between them. Approximate Bayesian Computation analysis suggested that the population size was stable in Bonin and Yaeyama + Daito groups, whereas population reduction occurred in Volcano group, and migration between groups were very limited. Younger oceanic islands showed lower genetic diversity, probably due to limited gene flow and a lack of time to accumulate unique alleles. Genetic structure was generally consistent with the geographic pattern of the islands, but in Volcano, a limited number of founders and limited gene flow among islands are likely to have caused the large genetic divergence observed.

## Introduction

Islands have long been important systems in ecology and evolutionary biology [1,2]. Due to the small size of their landmasses, isolation from source areas, simple biotas with relatively small number of species, and high levels of plant endemism, they provide excellent opportunities to investigate the evolutionary processes of plants. Islands are also suitable for studies of population genetics, to examine phenomena such as migration rates, degree of genetic isolation, and the extent of founder effects, since they are surrounded by water restricting the movement of terrestrial organisms [3]. The genetic diversity of plant populations on islands is likely to be influenced by island characteristics such as the geological origin of the island, its age, size, and distance to the nearest continental landmass. In the past decade, ecologists have applied island biogeography theory to investigations of genetic diversity, arguing that genetic and species diversity might be influenced by similar ecological processes [4]. For example, random extinctions of species in island communities are similar to the loss of alleles due to genetic drift [5], and thus factors influencing the species diversity of islands could influence the genetic diversity of the flora and fauna on islands. The genetic diversity of plant populations is generally lower on islands than in continental areas [6–8], and is lower on young islands than on old islands [9–11], although there are some exception [12,13]. Significant relationships have been found between genetic diversity and island area [14–17], and distance to the mainland [15,17–19]. Knowledge about genetic divergence within species, and its relationship to the island origin, age, size, and distance to the continent is essential to understanding the way in which plants colonize new islands and maintain genetic diversity.

**Fig 1:**
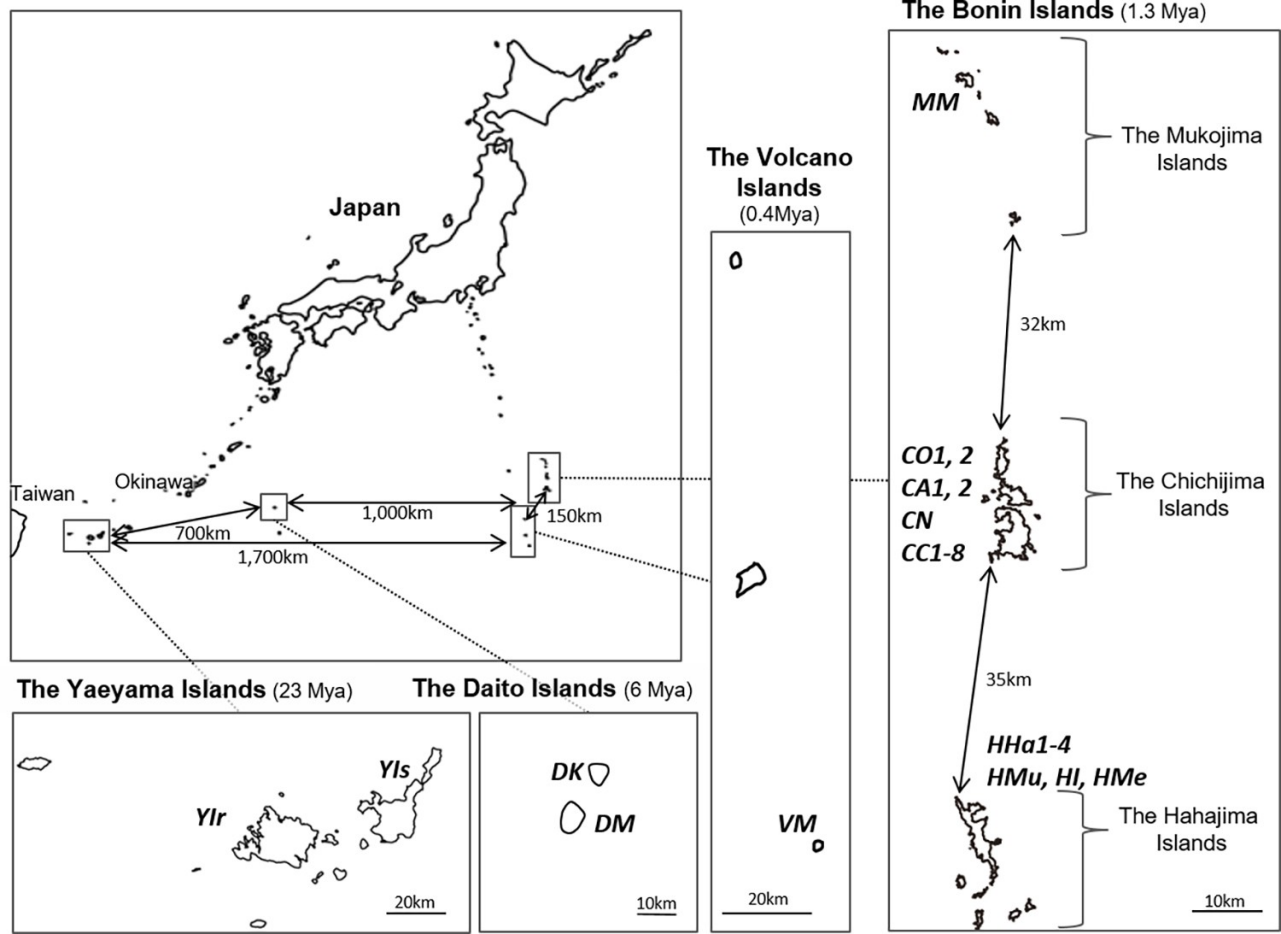
Location of the Bonin, Volcano, Yaeyama and Daito Islands. Bold italic letters show the Population ID of the sampled populations, shown in Table 1. Numbers in the parentheses are approximate age of islands used in the lmer analyses. Maps were drawn by ArcGIS using the coast line data downloaded from the Geographical Survey Institute of Japan (https://nlftp.mlit.go.jp/ksj/gml/datalist/KsjTmplt-C23.html), under a CC BY license, with permission from the Geographical Survey Institute of Japan, original copyright 2006.

Oceanic islands are defined as those which have never been connected to a continental landmass. They are the products of volcanism or tectonic uplift, or the results of organic reef growth upon foundations formed by the first two processes. Most continental islands were joined to other continental landmasses in the past, having since become separated due to tectonics or sea level rise [20]. In Japan there are oceanic and continental islands which have common subtropical climates, facilitating the comparison of genetic diversity between the islands. The Ogasawara Islands, including the Bonin and Volcano Islands, and the Daito Islands, are oceanic islands. However, the geneses of these islands differ, with the Ogasawara Islands being of volcanic origin while the Daito Islands are a result of uplifted atolls. The Yaeyama Islands are continental islands, which have been repeatedly connected to the Asiatic continent through Taiwan. The Bonin Islands are located 1,000 km south of mainland Japan in the Northwest Pacific Ocean, and include the Mukojima, Chichijima, and Hahajima Islands (Fig 1). They developed 44–34 mya [21,22], and were gradually uplifted before the middle Pleistocene [23]. The Volcano Islands are situated 150 km south of the Bonin Islands, and appeared 0.75–0.01 mya [24]. The Daito Islands, located about 360 km east of the mainland of Okinawa and about 1,000 km west of the Bonin Islands, are comprised of three small islands, Kitadaitojima, Minamidaitojima, and Okidaitojima. They developed on the sea bed 48 mya, sank under the sea 42 mya, and were uplifted again around 6 mya [25]. The Yaeyama Islands are located 400 km west of the mainland of Okinawa and 100 km east of the Taiwan. The South Ryukyu, where the Yaeyama Islands are located, developed between the late Paleozoic and the Mesozoic [26], and emerged around the Miocene (23 mya) [27,28]. Due to clear geological differences between these island groups, the genetic diversity, divergence and demography of plants are expected to differ between them.

*Planchonella obovata* sensu lato (Sapotaceae) is distributed in many parts of South-east Asia, south China, Taiwan, Micronesia, northeast Australia, and on islands of the Indian Ocean. In Japan, it occurs on the Ryukyu, Daito, Bonin, and Volcano Islands. *Planchonella obovata s*.*l*. is distributed widely from dry scrub to mesic forests on almost all of the Bonin Islands and from coastal areas to limestone areas in the mountains of the Ryukyu Islands, and is a major component of the natural vegetation. Thus, this species is ideal for the investigation of the genetic diversity, genetic structure, and population demography of plants in island areas of Japan.

The genus *Planchonella* includes around 110 species [29], and two species are found in Japan: *P*. *obovata s*.*l*. and *P*. *boninensis* (Nakai) Masam. et Yanagih. *Planchonella obovata s*.*l*. has two varieties, *P*. *obovata* (R.Br.) Pierre var. *obovata* and *P*. *obovata* var. *dubia* (Koidz. ex H.Hara) Hatus. ex T.Yamaz. *Planchonella obovata* var. *dubia* is distributed only on the Bonin and Daito Islands [30,31] where *P*. *obovata* var. *obovata* is also found. On these islands, *Planchonella obovata* var. *dubia*, inhabits dry rocky areas and is characterized by a lower stature and smaller leaves and fruits than *P*. *obovata* var. *obovata*, which grows in wetter habitats. However, the size of the leaves and fruits vary continuously between the two varieties, and are difficult to identify, and Ohashi and Kato [32] recognized *P*. *obovata* var. *dubia* as one of the ecotypes in dry areas.

In the Bonin Islands, adaptive radiation, probably caused by ecological divergence together with strong environmental gradients associated with the transition from mesic forests to dry scrub, has been observed in many genera such as *Callicarpa* [33], *Crepidiastrum*, *Pittosporum*, and *Symplocos* [34]. *Elaeocarpus photiniifolia*, endemic to the Bonin Islands, include genetically distinct groups associated with dry and mesic environments within the island [35]. Thus, the two varieties of *P*. *obovata* may be genetically distinct, although their phenotypic differences are slight.

The aim of this study was to answer following questions; (a) Are *P*. *obovata* var. *obovata* and *P*. *obovata* var. *dubia* genetically distinct? (b) What are the levels of population genetic diversity of *P*. *obovata s*.*l*., and which factors such as islands origin, age, area, and distance from the continent have the most impact on this diversity? (c) What is the *P*. *obovata s*.*l*. population structure in island areas in Japan? (d) Have the populations in each island group experienced past population size changes, such as expansion or reduction? (e) Is there migration among the island groups? The results of our study will contribute our understanding about colonization of continental plants on islands and how they maintain their genetic diversity.

## Materials and methods

### Study species and sample collection

*Planchonella obovata s*.*l*. is evergreen tree species, which is morphologically gynodioecious, although Kato et al. [36] have pointed out that it is functionally dioecious. Pollen is dispersed by insects, including flies, Oedemeridae beetle, and moths, which have been observed to visit flowers in the Bonin Islands [37,38]. Its berries are black, 1.2–1.5 cm long, and bear several 8–12 mm long seeds [30]. Primates, bats, lizards, and birds are thought to be important seed dispersers [39]. In the Bonin Islands, intact seeds are found in the feces of birds such as Japanese white-eye, Bonin Islands white-eye, and Brown-eared bulbul [40]. *Planchonella obovata s*.*l*. is distributed in both oceanic and continental islands, which vary in age, size, and distance to the continent, and is therefore appropriate for the investigation of the way in which genetic diversity is maintained in the islands.

We collected leaf samples of 663 individuals of *P*. *obovata s*.*l*. from 28 populations, 14 islands of the Bonin, Volcano, Daito, and Yaeyama Islands (Fig 1, Table 1). To ascertain whether *P*. *obovata* var. *obovata* and *P*. *obovata* var. *dubia* can be discriminated genetically, we sampled both where they occur in close proximity from the same location on Chichijima island (population CC4). These consisted of 17 individuals from dry rocky areas of typical *P*. *obovata* var. *dubia*, which is short in height with small leaves (CC4_dubia), and 35 individuals from wetter forest areas of typical *P*. *obovata* var. *obovata*, which is tall in height with large leaves (CC4). In the Daito Islands, there should be *P*. *obovata* var. *dubia* according to some previous reports, but we could not find typical *P*. *obovata* var. *dubia* there. After collection, the leaf samples were dried with silica gel. We recorded the locations of the sampled individuals using a GPS (GPSmap60CSx; Garmin, Olathe, Kansas, USA). Voucher specimens of each population were deposited in the herbarium of the Forestry and Forest Products Research Institute, Japan (nos. TF-FDA001379–TF-FDA001431) and Makino Herbarium, Tokyo Metropolitan University, Japan (nos. MAK378903, 378904, 391087).

**Table 1:** Population genetic parameters estimated from 11 SSR for the 27 *Planchonella obovata* var. *obovata* and one *P*. *obovata* var. *dubia* population (CC4_dubia).

Island groups		Islands	Island area(ha)	Population ID	No. of samples	*A*_R_	*H*_O_	*H*_E_	*F*_IS_
The Bonin Islands									
	The Mukojima Islands	Mukojima	256	MM	32	4.37	0.57	0.58	0.03
	The Chichijima Islands	Otoutojima	520	CO1	22	4.87	0.59	0.57	0.00
				CO2	39	5.01	0.58	0.60	0.05
		Anijima	788	CA1	44	4.68	0.57	0.59	0.05
				CA2	29	4.84	0.55	0.59	0.09
		Nishiijima	48	CN	12	4.15	0.54	0.52	0.02
		Chichijima	2344	CC1	20	4.79	0.56	0.57	0.05
				CC2	21	4.36	0.52	0.55	0.09
				CC3	17	4.50	0.50	0.53	0.09
				CC4	35	4.88	0.54	0.58	0.09
				CC4_dubia	17	4.71	0.55	0.56	0.05
				CC5	24	4.93	0.52	0.57	0.12
				CC6	20	4.74	0.56	0.57	0.04
				CC7	24	4.53	0.55	0.58	0.07
				CC8	21	4.50	0.57	0.55	0.00
	The Hahajima Islands	Hahajima	1988	HHa1	23	4.54	0.56	0.59	0.07
				HHa2	23	4.16	0.57	0.58	0.05
				HHa3	23	4.35	0.59	0.59	0.03
				HHa4	20	4.85	0.66	0.62	-0.04
				HHa5	25	4.38	0.62	0.62	0.03
		Mukoujima	138	HMu	20	4.71	0.56	0.62	0.12
		Imoutojima	123	HI	10	4.36	0.48	0.57	0.20
		Meijima	87	HMe	23	4.49	0.60	0.59	0.00
The Volcano Islands									
		Minamiiwoto	354	VM	35	3.17	0.47	0.51	0.09
The Daito Islands									
		Kitadaitojima	1194	DK	26	4.24	0.58	0.59	0.04
		Minamidaitojima	3057	DM	23	4.03	0.48	0.50	0.07
The Yaeyama Islands									
		Ishigakijima	22250	YIs	14	5.28	0.58	0.65	0.13
		Iriomotejima	28930	YIr	21	5.69	0.72	0.72	0.03

*A*_*R*_; allelic richness, *H*_O_; observed heterozygosity, *H*_E_; gene diversity, *F*_IS_; fixation index.

All *F*_IS_ values were not significantly deviated from Hardy-Weinberg equilibrium.

### Microsatellite analysis

Total genomic DNAs of all sampled leaves were extracted, using DNeasy Plant Mini Kits (QIAGEN, Hilden, Germany). Eighteen nuclear microsatellite markers were developed by one *P*. *obovata* var. *obovata* plant from Hahajima in the Bonin Islands, and details of marker development was described in S1 Appendix and S1 Table in S1 File. We genotyped all 663 samples using 18 primer pairs, with the experimental conditions described in S1 Appendix in S1 File. We tested the existence of null alleles using Microchecker [41], and the linkage disequilibrium between loci in each population using FSTAT 2.9.3.2 [42].

Five (Po124, Po200, Po281, Po579, and Po583) out of 18 markers might have null alleles since estimated null allele frequency was significant in more than five populations. Two other markers, Po290 and Po623, were characterized by large amounts of missing data. No significant linkage disequilibrium was observed between loci in any population for the 11 markers, excluding the above mentioned seven markers. Thus, we used 11 markers for further population genetic analyses. Microsatellite genotype data for 11 markers are available in S1 Data.

### Data analysis

#### Statistical analyses of genetic diversity

To evaluate the genetic diversity of each population, allelic richness (*A*_R_) [43], observed heterozygosity (*H*_O_) and gene diversity (*H*_E_) [44] were calculated for each locus and each population using GenAlEx ver. 6.501 [45]. The fixation index (*F*_IS_) was calculated and tested by randomization using FSTAT ver. 2.9.3 [46]. We used linear mixed-effect models (lmer) to examine the associations between genetic diversity (*A*_R_, *H*_E_) within each population and island characteristics such as its island age, area, origin (oceanic or continental), and distance to mainland China using the R package lme4 [47]. The island age (1: young; Volcano, 2: middle; Bonin and Daito, 3: old; Yaeyama), area (log-transformed), origin (1: oceanic, 0: continental), and distance to nearest continent were treated as fixed effects, and the differences between loci as random effects. Island area was log-transformed to reduce skewness and increase the normality of its distribution. Variables were checked for collinearity using the variance inflation factor (VIF) value, which should be less than 5 [48], using the R package car [49]. The VIF for island origin was 5.11. We eliminated the island age from the lmer analysis, since island age and origin were highly correlated. We also conducted lmer analysis omitting the data of continental island populations, to eliminate the effect of island origin. The island age (0: young, 1: middle), area (log-transformed), and distance to nearest continent were treated as fixed effects, and the differences between loci as random effects. Data for *H*_E_ were arcsine-transformed to obtain closer approximations to normality. Eight candidate models with 0–3 fixed explanatory variables were constructed for each response variable. We calculated Akaike’s information criterion to evaluate the candidate models (AIC) [50] values for each of them. The differences between the AIC values and the minimum AIC value (ΔAIC) were calculated for each of the models, and models with ΔAIC value ≤ 2 were selected as the best models [51]. The analyses were conducted using R software 4.0.2 [52]. For the lmer analysis, data from individuals sampled as *P*. *obovata* var. *dubia* in population CC4_dubia were merged with population CC4, since there was no genetic difference between the two populations (see details in Results). Data used in the lmer analysis are available in S2 Data.

#### Statistical analyses of genetic structure

For population genetic structure analysis, we firstly conducted Bayesian clustering analysis using 35 typical *P*. *obovata* var. *obovata* and 17 typical *P*. *obovata* var. *dubia* individuals from the Bonin Islands, and 49 samples from the Daito Islands, to check whether *P*. *obovata* var. *dubia* was genetically different from *P*. *obovata* var. *obovata*, using the program STRUCTURE 2.3.4 [53,54]. In this analysis, we chose allele frequency correlated model and admixture model to detect the admixture of lineages, and each run involved 100,000 Markov chain Monte Carlo (MCMC) iterations after a burn-in period of 50,000 iterations. The analysis was run 30 times with each *K*, ranging from 1 to 10. Then, the population genetic structure of all *P*. *obovata s*.*l*. populations were investigated using STRUCTURE, reducing the sample size of the Chichijima and Hahajima Islands populations to 45 randomly selected individuals each, since the program may show a bias when the sampling design is unbalanced [55]. In this analysis, each run involved 100,000 MCMC iterations after a burn-in period of 50,000 iterations. The analysis was run 30 times with each *K*, ranging from 1 to 15. The optimal value for *K* was evaluated using the Δ*K* [56] and the mean log likelihood at each *K* [54]. STRUCTURE tends to detect the highest level of a population hierarchy [56], and there can be lower hierarchy of structuring within the highest clusters. Thus, we also explored the substructure within each detected cluster. Results were summarized using the CLUMPAK [57].

The genetic relationships among populations were assessed by a neighbor-joining (NJ) tree based on the *D*_A_ genetic distance [46] between them, using the program Populations 1.2.32 [58]. The significance of each node in the tree was evaluated by 1,000 bootstraps. The isolation by distance (IBD) [59] pattern was evaluated by the Mantel test [60] on population pairwise natural logarithms of geographical distance (ln (1 + geographical distance)) and *F*_ST_/(1 –*F*_ST_) [61].

#### Statistical analyses of population demography

**Fig 2:**
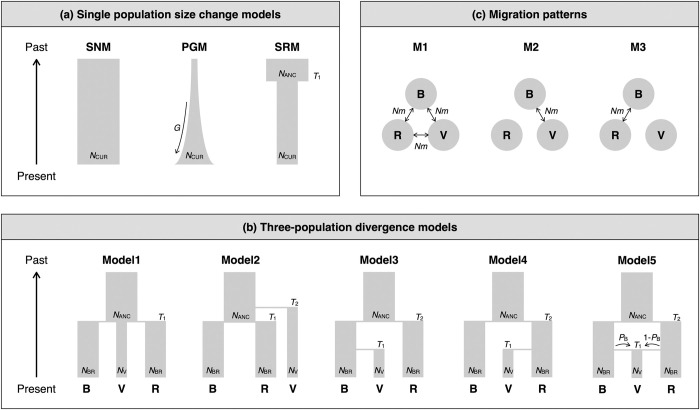
Population demographic models. Three single population size change models (a; SNM, standard neutral model; PGM, population growth model; SRM, size reduction model). Five three-population divergence models (b; B, Bonin group; V, Volcano group; Y+D, Yaeyama and Daito group). Three migration patterns (c).

STRUCTURE analysis detected clear genetic structure among three island groups: the Bonin Islands; the Volcano Islands; and the Yaeyama and Daito Islands (see details in Results). In order to estimate the population demography of these three island groups, we conducted approximate Bayesian computation (ABC). As there are various patterns in combinations of population demography—population size change, population divergence, and migration patterns—we sequentially executed ABC analyses [11,62]. In the first step, we applied single population size change models for each island group (Fig 2A). In the second step, we applied three-population divergence models without migration for the three island groups (Fig 2B). Finally, in the third step, we examined divergence models with and without migration (Fig 2C). The detail of ABC analysis was described in S2 Appendix in S1 File.

## Results

### Genetic difference between *P*. *obovata* var. *obovata* and *P*. *obovata* var. *dubia*

**Fig 3:**
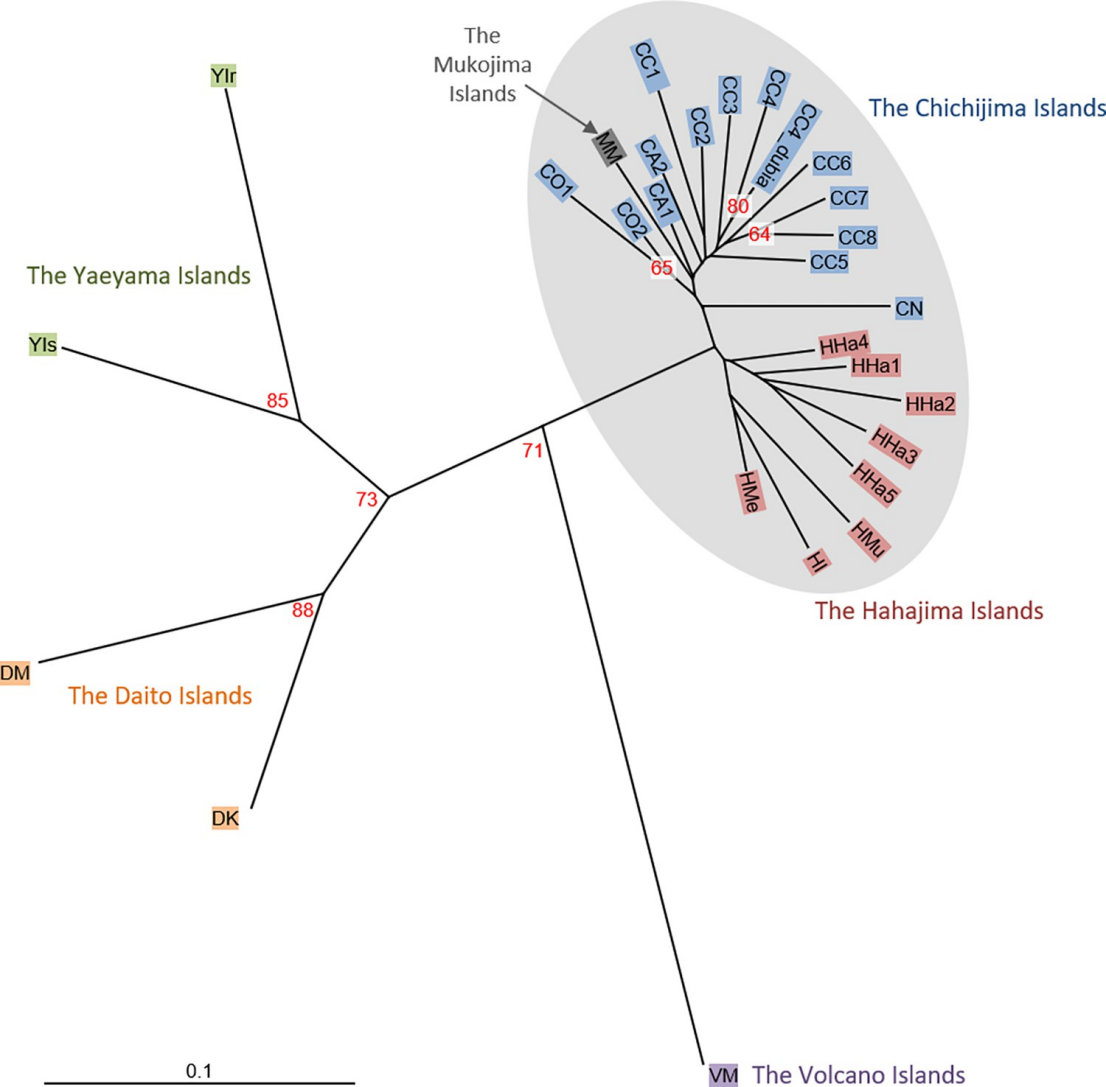
A neighbor-joining dendrogram for 27 *Planchonella obovata* var. *obovata* and one *P*. *obovata* var. *dubia* populations based on 11 SSR markers based on genetic distance, *D*_A_. Numbers in the internal nodes indicate bootstrap value larger than 50; gray oval, populations in the Bonin Islands.

**Fig 4:**
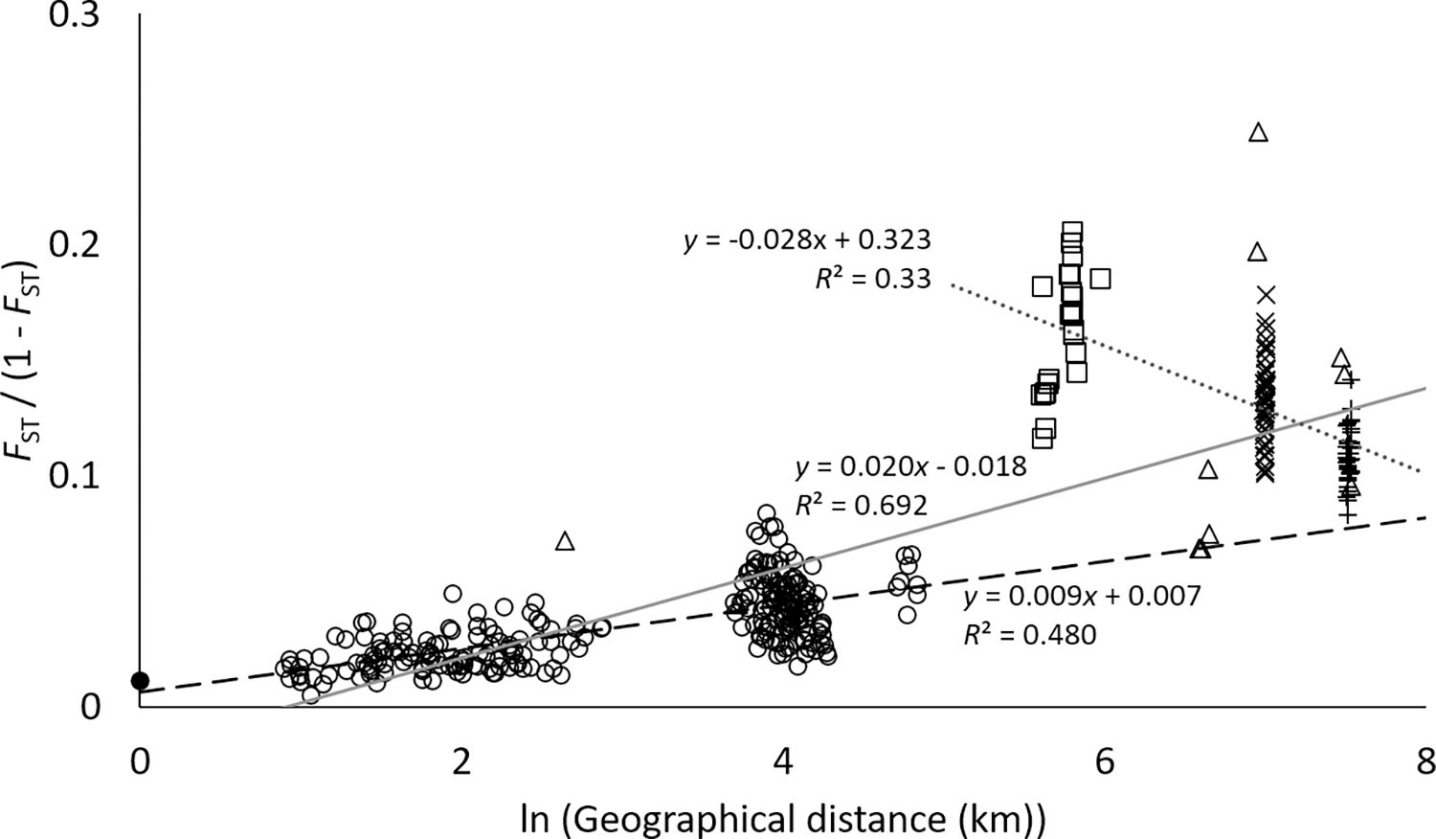
Relationship between pairwise genetic differentiation and natural logarithms of geographical distance for all the 27 *P*. *obovata* var. *obovata* and one *P*. *obovata* var. *dubia* populations. Solid circle, population pair between *P*. *obovata* var. *obovata* (CC4) and *P*. *obovata* var. *dubia* (CC4_dubia); open circles, population pairs within the Bonin Islands; squares, population pairs between the Bonin and Volcano Islands; cross signs, population pairs between the Bonin and Daito Islands; plus signs, population pairs between the Bonin and Yaeyama Islands; triangles, population pairs between other islands; solid gray line, regression line for all 28 populations; black broken line, regression line for population pairs within the Bonin Islands; gray dotted line, regression line for population pairs whose geographical distance over 270 km (ln (1 + geographical distance > 5.6)).

According to a NJ tree based on the *D*_A_ genetic distance, the *P*. *obovata* var. *obovata* (CC4) and *P*. *obovata* var. *dubia* (CC4_dubia) sampled from the same site on Chichijima island in the Bonin Islands, formed a cluster with a high bootstrap value (Fig 3). The pairwise *F*_ST_ between CC4 and CC4_dubia was low as 0.012 (Fig 4). STRUCTURE analysis showed no genetic differentiation between CC4 and CC4_dubia with increasing *K* (S1 Fig in S1 File). In the Daito Islands, there was genetic differentiation between the two sampled populations, Kitadaitojima and Minamidaitojima, however no clear genetic sub-structuring which would imply the existence of *P*. *obovata* var. *dubia* was found within populations.

### Genetic diversity

**Fig 5:**
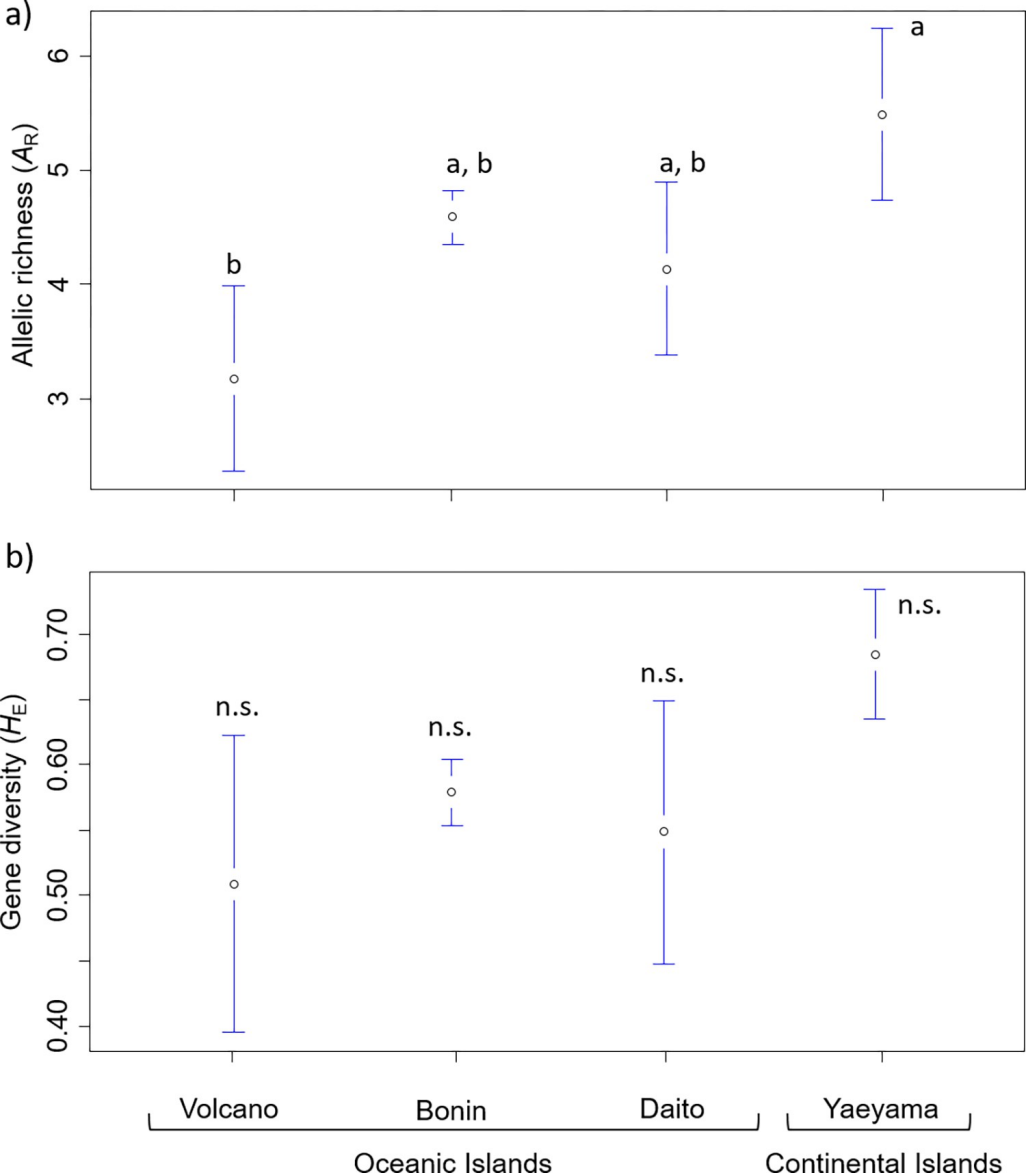
Mean allelic richness (*A*_R_) and gene diversity (*H*_E_) in the four islands of *Planchonella ovobata* (± SE). Different letters indicate significant differences among islands (*p* < 0.05, pairwise t-test with Bonferroni correction). Island groups are ordered from youngest (left) to oldest (right).

At the 11 SSR loci examined, allelic richness (*A*_R_) ranged from 3.17–5.69 (mean 4.58), and gene diversity (*H*_E_) ranged from 0.50–0.72 (mean 0.58, Table 1). The *A*_R_ values of each island group were significantly different (Kruskal–Wallis test, *p* < 0.01). The *A*_R_ values of the Yaeyama Islands were significantly higher than that of the Volcano Islands (pairwise t-test, *p* < 0.01, Fig 5A), and those of the Yaeyama and Daito, and Bonin and Volcano Islands were marginally significant (pairwise t-test, *p* = 0.08, 0.07, respectively). The *H*_E_ values of each island group were significantly different (Kruskal–Wallis test, *p* < 0.05), and the *H*_E_ of the Yaeyama and Volcano islands was marginally significant (pairwise t-test, *p* = 0.08, Fig 5B).

In the lmer analysis investigating the associations between genetic diversity and island characteristics for all populations, we eliminated island age from the analysis, since island origin and age were highly correlated. For the lmer analysis explaining *A*_R_ and *H*_E_, the best model both consisted only of island origin (S2 Table in S1 File), and the coefficients of island origin were negative for both *A*_R_ and *H*_E_ (S3 Table in S1 File). To eliminate the effect of island origin, we also conducted lmer analysis using only oceanic islands data. For the analysis explaining *A*_R_, the best model included only island age. For the analysis explaining *H*_E_, the null model had the smallest AIC, while the Δtheilet AICsecond-best model was ≤ 2, and included island age. Coefficients of island age were positive for both *A*_R_ and *H*_E_.

### Genetic structure

In an NJ tree based on genetic distance, *D*_A_, *P*. *obovata s*.*l*. populations clustered into four distinct groups: the Bonin, Volcano, Daito, and Yaeyama Islands with high bootstrap values (Fig 3). In the Bonin Islands, populations were grouped into two: the Mukojima and Chichijima Islands, and the Hahajima Islands. The Daito Islands were closer to the Yaeyama Islands than to the Bonin Islands. The Volcano Islands population was located between the Bonin Islands and the Yaeyama and Daito Islands, with a long branch.

**Fig 6:**
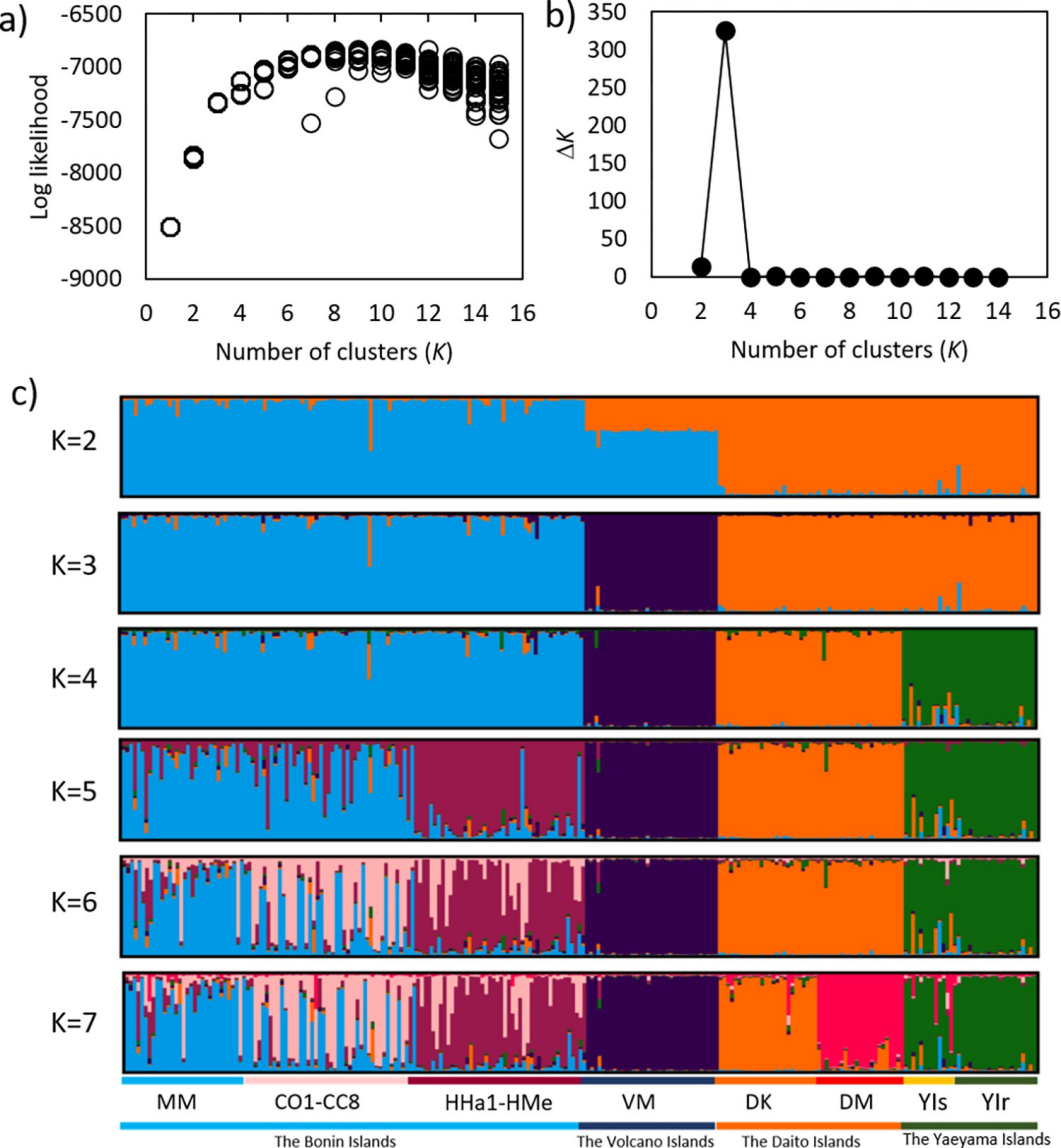
Results of STRUCTURE analysis for all *Planchonella obovata s*.*l*. populations based on 11 SSR markers. Forty-five samples from the Chichijima and Hahajima Islands were selected at random. Changes in loglikelihood and Δ*K* as the number of clusters (*K* ranging from 1 to 15), barplots of 241 genotypes at *K* = 2 to 9. Vertical columns represent individuals; heights of bars are proportional to the posterior means of the estimated admixture proportions.

STRUCTURE analysis for all *P*. *obovata s*.*l*. populations showed that the Δ*K* was highest when *K* = 3: the Bonin Islands; the Volcano Islands; and the Yaeyama and Daito Islands were separated (Fig 6). At *K* = 4, the Daito and Yaeyama islands were separated. At *K* = 5, the Mukojima (MM) and Chihijima Islands (CO1-CC8), and the Hahajima Islands (HHa1-HMe). At *K* = 6, the Mukojima and Chihijima Islands were separated. At *K* = 7, the log likelihood reached highest value, Kitadaito (DK) and Minamidaito Islands (DM) were separated.

There were significant IBD among all populations (*R*^2^ = 0.692, *p* < 0.05, Fig 4) and among populations in the Bonin Islands (*R*^2^ = 0.480, *p* < 0.01), and regression coefficients were higher among all populations (all populations = 0.020; the Bonin Islands = 0.009). On the other hand, significant negative correlation was detected between geographical distance and pairwise *F*_ST_ among populations whose geographical distance over 270 km (ln (1 + geographical distance) > 5.6, *r* = -0.577, *p* < 0.001). Higher pairwise *F*_ST_ values were observed between the populations of the Bonin and Volcano Islands.

### Population demography and divergence

The best of the three single population size change models, as selected by ABC-RF, were the standard neutral model (SNM) in the Bonin and Yaeyama + Daito groups, and the size reduction model (SRM) in the Volcano group, with relatively low error rates (0.255–0.279) and high posterior probabilities (0.757–0.791; Fig 2A, S4 Table in S1 File). Using the best models, we estimated the posterior distribution of parameters. All parameters, except for the relative ancestral effective population size (*RN*_ANC_) in log_10_ scale, and the mean geometric parameter in the generalized stepwise mutation model (*P*_GSM_) in the SRM of the Volcano Island group, showed a clear single peak (S2 Fig in S1 File). The Bonin and Yaeyama + Daito groups showed similar level of current effective population size (*N*_CUR_) and its posterior modes (95% HPD) were 10,436 (6,517–18,411) and 12,468 (7,283–23,472), respectively, while Volcano had a smaller value of *N*_CUR_ than the others, at 1,829 (401–4,561; S4 Table in S1 File). Most of the summary statistics predicted by the posterior distribution fell near the observed values, and we concluded that the goodness-of-fit values of the single population size change models to the observed data were high (S3 Fig in S1 File).

In a comparison of the five three-population divergence models, Model3 was the best model, with a posterior probability of 0.431 (Fig 2B, Table 2). However, votes of Model1 (0.265) was not low compared with those of Model3 (0.369), and the error rate was also relatively high (0.388). This high error rate was due to the high classification error rate of Model5, as shown in the confusion matrix (0.646; S5 Table in S1 File). Thus, we removed Model5 and compared the remaining four models. In this comparison, although Model3 was again selected as the best model, and the error rate was reduced (0.295), the posterior probability was still not high (0.516), and the votes of Model1 were also still not low (0.352) compared to those of Model3 (0.429) (Table 2). As the votes for the other two models were low (0.117 for Model2 and 0.102 for Model4), we removed these two models and compared only Model1 and Model3. In this comparison, Model3 was again selected as the best model, and had a high posterior probability (0.752) and a low error rate (0.184) (Table 2). Therefore, we confirmed that Model3 was the best model among the five three-population divergence models. Using the best divergence model, Model3, we compared models with and without migration using ABC-RF, and the without migration model was strongly supported, having a posterior probability of 0.954 (Fig 2C, Table 2). We thus estimated the posterior distribution of the parameters in Model3, and all parameters showed a clear single peak (S4 Fig in S1 File). The posterior mode (95% HPD) of the current effective population sizes in the Bonin and Yaeyama + Daito groups (*N*_BYD_), and the Volcano group (*N*_V_) were 15,213 (8,743–29,273) and 1,710 (690–4,537), respectively (S6 Table in S1 File). The posterior mode (95% HPD) of the time of divergence of the Volcano group from the Bonin group (*T*_1_) was 1,623 (295–5,169) generations ago. That between the Bonin and the Yaeyama + Daito groups (*T*_2_) was 8,652 (2,888–21,509) generations ago. Most of the summary statistics predicted by the posterior distribution fell near the observed values, and we concluded that the goodness-of-fit of Model3 to the observed data was high (S5 Fig in S1 File).

**Table 2:** Proportion of votes by random forest, posterior probability of the best model, and classification error rate in population divergence and migration analyses.

	Proportion of votes ^a^					Posterior	Classification
Compared model set	Model1	Model2	Model3	Model4	Model5	probability	error rate
Five divergence models	0.265	0.096	**0.369**	0.068	0.202	0.431	0.388
Four divergence models	0.352	0.117	**0.429**	0.102	—	0.516	0.295
Two divergence models	0.366	—	**0.634**	—	—	0.752	0.184
	Model3M1	Model3M2	Model3M3	Model3			
With/without migration models	0	0.004	0.018	**0.978**		0.954	0.216

^a^ Best model is shown in bold.

## Discussion

### *P*. *obovata* var. *obovata* and *P*. *obovata* var. *dubia*

We could not differentiate *P*. *obovata* var. *obovata* and *P*. *obovata* var. *dubia* samples collected in the Bonin Islands genetically using either a NJ tree or STRUCTURE analysis. In the Daito Islands, no genetic sub-structuring which would imply the existence of *P*. *obovata* var. *dubia* were found within populations using the STRUCTURE analysis. Thus, we concluded that *P*. *obovata* var. *dubia* is one of the phenotypic variations of *P*. *obovata* var. *obovata* in this study. However, considering that the leaves of the individuals we sampled from the Daito Islands were larger than those of specimens identified as *P*. *obovata* var. *dubia* in the Daito Islands (nos. RYU4937 and RYU4955), there is a possibility we did not sample genuine *P*. *obovata* var. *dubia* there. More intensive search for typical *P*. *obovata* var. *dubia* and research combining genetic data and phenotypic data such as the sizes of leaves and fruits should be conducted in the Daito Islands in the future.

### Genetic diversity of *Planchonella obovata* sensu lato

Allelic richness (*A*_R_) was highest in the Yaeyama Islands (mean 5.49), moderate in the Bonin and Daito Islands (means 4.60 and 4.14, respectively), and lowest in the Volcano Islands (3.17). Gene diversity (*H*_E_) showed the same pattern as *A*_R_, being highest in the Yaeyama Islands (mean 0.68), moderate in the Bonin and Daito Islands (means 0.58 and 0.55, respectively), and lowest in the Volcano Islands (0.51). This is consistent with previous studies with Maki [63] finding that allozyme diversity of plants endemic to the continental Ryukyu Islands (*H*_T_ 0.134–0.321) are about five times higher than plants endemic to the oceanic Bonin Islands (*H*_T_ 0.000–0.0083). In addition, using microsatellites, lower genetic diversity in the Volcano Islands compared with the Bonin Islands were also observed in *Pandanus boninensis*, an endemic species in the Bonin and Volcano Islands [11].

The lmer analyses suggested that island origin and age contributed most to the *A*_R_ and *H*_E_ levels. Island origin affected the genetic diversity, with populations in the continental islands having higher genetic diversity than oceanic ones. This is probably due to difference in number of founders with populations on oceanic-islands being established from limited seeds arriving via rare long-distance dispersal. In contrast, populations on continental-islands should have experienced greater seed influx during and post- establishment through seed dispersal by birds and other animals in the past when the continental islands were connected to continental landmasses. Island age also affected the genetic diversity, with populations on older islands having higher genetic diversity. This observation is consistent with studies in other island groups, such as the Canary Islands [9] and New Caledonia [10]. This phenomenon probably arises because unique alleles have not had enough time to accumulate via mutation and recombination on younger islands [64,65]. However, island area and distance to nearest continent did not contribute to the observed differences in genetic diversity. Although, higher genetic diversity in large populations is usually observed [66], and in the Bonin Islands, a positive correlation between genetic diversity and island area has been found in *Pa*. *boninensis* [11], in *P*. *obovata s*.*l*., island size may not be a good indicator of population size since its distribution is restricted to the coastal area especially in the Yaeyama Islands. Interestingly, distance to nearest continent did not impact genetic diversity probably because geographical distance used in lmer were too large to test the effect on the genetic diversity of *P*. *obovata s*.*l*. This is likely due to the fact that migration among island groups is extremely low, as evidenced by Model3 without migration being selected in the population divergence analysis, and, although overall isolation by distance was significant, a significant negative correlation for the population pairs whose distance over 270 km was observed, suggesting no isolation by distance pattern over this geographical distance. Given that the shortest distance to nearest continent in our data is 450 km (the Yaeyama Islands) recent migration into investigated islands from continental areas would also very limited and might have no effects on the genetic diversity.

#### Genetic structure of *Planchonella obovata* sensu lato

NJ tree and STRUCTURE analysis of all *P*. *obovata s*.*l*. populations identified three genetic groups: the Bonin Islands; the Volcano Islands; and the Yaeyama and Daito Islands. This finding was broadly consistent with the geographic arrangement of the islands. The Yaeyama and Daito Islands populations were adjacent in the NJ tree, and they were in the same cluster in the STRUCTURE analysis when *K* = 4, suggesting that the younger Daito Islands populations may have diverged from the older Ryukyu Islands where the Yaeyama Islands are located. This result is plausible, because most of the plants in the Daito Islands are shared with the Ryukyu Islands [67]. The Volcano Islands population was equally distant from the Bonin and Yaeyama + Daito Islands populations in the NJ tree, and STRUCTURE analysis could not determine which genetic groups were close to the Volcano Islands (Fig 6C, *K* = 2, 3). Population divergence analysis was able to unravel this question. Model3, in which the Bonin and Yaeyama + Daito groups diverged from the ancestral population, and then the Volcano group diverged from the Bonin group, was selected as the best model. The younger Volcano Islands population was inferred as having diverged from the older Bonin Islands populations. This pattern was also found in *Pa*. *boninensis* in the same area [11], and other islands, *Plantago* in the Hawai’ian Islands [68] and *Drimys* in the Juan Fernandez archipelago [69]. We only sampled *P*. *obovata s*.*l*. in Japan, and other candidates, such as Northern Mariana Islands, 540 km south of the Volcano Islands, were not included in our analyses. However, if the Mariana Islands is a source population of the Volcano Islands, Model1 or 2 should have been selected by ABC-RF. We should therefore include these islands to correct the estimation of the origins of the populations in the future.

The results of NJ tree, STRUCTURE analysis, and IBD analysis indicated populations in the Bonin and Volcano Islands were more distinct than may be expected given the relatively short geographical distance between them. This is probably caused by the following two factors. One is the founder effect would have occurred in the Volcano Islands since the size reduction model (SRM) was selected from population size change analysis in the Volcano Island group. This finding suggests that the number of founders which migrated from the Bonin Islands to the Volcano Islands was very limited. The other is gene flow from the Bonin Islands to the Volcano Islands was very limited as described above, and thus increased the genetic divergence between them.

In the Bonin Islands, *P*. *obovata s*.*l*. populations were clustered into two genetic groups: the Mukojima and Chichijima Islands, and the Hahajima Islands using NJ tree and STRUCTURE analysis with *K* = 5. This genetic pattern is also found in other flora and fauna dispersed by ocean currents in the Bonin Islands, such as *Terminalia catappa* [70], *Pa*. *boninensis* [11], *Hibiscus* [71], lizards [72] and land snails [73]. Fruits of *P*. *obovata s*.*l*. consist of a black berry and are thought to be adapted to dispersal by birds. Intact seeds of *P*. *obovata s*.*l*. were found in feces of Japanese white-eyes, Bonin Islands white-eyes, and Brown-eared bulbuls in the Bonin Islands [40]. Brown-eared bulbuls have high mobility, however, they are unlikely to disperse seeds between the Mukojima and Chichijima Islands, which are separated by 32 km, or the Chichijima and Hahajima Islands, which are 35 km apart, according to the retention time in the guts of the Brown-eared Bulbul, which is 30 min at most for 9.3 mm seeds [74] and their cruising speed of 29–36 km/h, calculated from body weight [75]. However, Swenson *et al*. [76] reported that *Planchonella* was dispersed as far as 8,900 km between Palau in the Pacific and the Seychelles in the Indian Ocean, based on a maximum clade credibility tree. Seeds of *P*. *obovata s*.*l*. are resistant to sea water [77]. Similar genetic patterns have been observed in ocean-distributed species, which have the potential to be dispersed over very long distances by water, and the salt tolerance of the seeds suggests that seeds would be dispersed by ocean currents among the Mukojima, Chichijima, and Hahajima Islands. This phenomenon whereby seeds are dispersed by vectors different from those to which they are best suited is called non-standard mechanisms of dispersal [78], and could play a role in the long distance dispersal of this species, resulting in island colonization [79].

### Population demography and divergence times

In the population size change analysis, the SNM was selected in the Bonin, Yaeyama + Daito groups, and the SRM was selected in the Volcano group. These results suggest that the population size was stable in the Bonin, Yaeyama and Daito Islands, while population reduction occurred in the Volcano Islands. A similar analysis was conducted for *Pa*. *boninensis* in the Bonin and Volcano Islands, and the population growth model (PGM) was selected in the Bonin Islands, while the SRM was selected for the Volcano Islands [11]. The SRM was selected in the Volcano Islands for both species, suggesting that the effect of a founder event in the young islands is still detectable. The divergence time of the Bonin and Yaeyama + Daito groups from the ancestral population (*T*_2_) for *P*. *obovata s*.*l*. was 8,652 generations ago, while that of Bonin from the ancestral population for *Pa*. *boninensis* was 91,925 (S4 Table in S1 File in Setsuko *et al*. [11]), and *P*. *obovata s*.*l*. is one digit younger than *Pa*. *boninenisis*, assuming that the generation time of *P*. *obovata s*.*l*. and *Pa*. *boninensis* is almost the same. This finding is consistent with the fact that the *Pa*. *boninensis* is endemic to the Bonin and Volcano Islands, so a sufficient amount of time has passed for the ancestral *Pandanus* to have speciated into an endemic species. In contrast, the colonization of *P*. *obovata s*.*l*. in the Bonin Islands is likely to be relatively recent, and colonization of the islands already occupied by other plant species, may prevented the species from undergoing an increase in population size as *Pa*. *boninenisis* did, and thus the SNM would be selected in the population size change analysis. However, population demography and divergence time estimates of *P*. *obovata s*.*l*. in this study were derived from only limited number of SSR markers using simple ABC approaches, and further investigation using markers with higher resolution such as genome wide SNPs and other statistical approaches should be undertaken in the future.

## Conclusion

We examined the genetic diversity, structure, and population demography of *P*. *obovata s*.*l*. on both continental (the Yaeyama Islands) and oceanic islands (the Daito, Bonin, and Volcano Islands) using 11 microsatellite markers. We could not differentiate *P*. *obovata* var. *obovata* and *P*. *obovata* var. *dubia* genetically, and concluded that *P*. *obovata* var. *dubia* is part of the phenotypic variation found in *P*. *obovata* var. *obovata*. Island origin and age had significant effects on the genetic diversity of *P*. *obovata s*.*l*. Genetic diversity was higher in the old continental islands (the Yaeyama Islands) and lower in the young oceanic islands (The Volcano Islands). This difference was probably caused by two reasons. One is difference in number of founders, which is greater on continental islands, and the other is difference of time to accumulate the new alleles by mutation and recombination. Genetic structure was generally consistent with the geographic pattern of the islands, but in the young oceanic islands, a limited number of founders and limited gene flow among islands is likely to have caused the large genetic divergence observed. ABC analysis revealed population size was stable in the old continental and older oceanic islands (the Bonin Islands), while population reduction occurred in the young oceanic islands, migration among the island groups were very limited, and suggested that the young oceanic islands were colonized by geographically close, older oceanic islands. Results of our study provides a good example about colonization of continental plants on islands and how they maintain their genetic diversity.

## Supporting information

S1 File (DOCX)

S1 Data Microsatellite genotype data for 11 markers. (XLSX)

S2 Data Data used in the lmer analysis. (XLSX)

## References

[1] MacArthur RH, Wilson EO. The theory of island biogeography. Princeton, UK: Princeton University Press; 2001.

[2] Darwin C. The structure and distribution of coral reefs. London, UK: Smith Elder and Co; 1842.

[3] Franks SJ. Genetics, evolution, and conservation of island plants. J Plant Biol. 2010; 53(1): 1–9. doi: 10.1007/s12374-009-9086-y

[4] Vellend M, Orrock JL. Ecological and genetic models of diversity. The theory of Island biogeography revisited New Jersey, USA: Princeton University Press; 2009. pp. 439–62.

[5] Vellend M, Geber MA. Connections between species diversity and genetic diversity. Ecol Lett. 2005; 8(7): 767–81. 10.1111/j.1461-0248.2005.00775.x.

[6] Duryea M, Zamudio K, Brasileiro C. Vicariance and marine migration in continental island populations of a frog endemic to the Atlantic Coastal forest. Heredity (Edinb). 2015; 115(3): 225–34. doi: 10.1038/hdy.2015.31 25920672 PMC4814237

[7] Crawford DJ, Ruiz E, Stuessy TF, Tepe E, Aqeveque P, Gonzalez F, et al. Allozyme diversity in endemic flowering plant species of the Juan Fernandez Archipelago, Chile: ecological and historical factors with implications for conservation. Am J Bot. 2001; 88(12): 2195–203. 21669652

[8] Hufford KM, Mazer SJ, Hodges SA. Genetic variation among mainland and island populations of a native perennial grass used in restoration. AoB PLANTS. 2014; 6: plt055. doi: 10.1093/aobpla/plt055 24790118 PMC3966692

[9] Saro I, González-Pérez MA, García-Verdugo C, Sosa PA. Patterns of genetic diversity in *Phoenix canariensis*, a widespread oceanic palm (species) endemic from the Canarian archipelago. Tree Genet Genomes. 2014; 11(1): 815. doi: 10.1007/s11295-014-0815-0

[10] Bottin L, Verhaegen D, Tassin J, Olivieri I, Vaillant A, Bouvet JM. Genetic diversity and population structure of an insular tree, *Santalum austrocaledonicum* in New Caledonian archipelago. Mol Ecol. 2005; 14(7): 1979–89. doi: 10.1111/j.1365-294X.2005.02576.x 15910320

[11] Setsuko S, Sugai K, Tamaki I, Takayama K, Kato H, Yoshimaru H. Genetic diversity, structure, and demography of *Pandanus boninensis* (Pandanaceae) with sea drifted seeds, endemic to the Ogasawara Islands of Japan: Comparison between young and old islands. Mol Ecol. 2020; 29(6): 1050–68. doi: 10.1111/mec.15383 32048374

[12] Curto M, Puppo P, Kratschmer S, Meimberg H. Genetic diversity and differentiation patterns in Micromeria from the Canary Islands are congruent with multiple colonization dynamics and the establishment of species syngameons. BMC Evol Biol. 2017; 17(1): 1–16. 28830342 10.1186/s12862-017-1031-y PMC5568322

[13] Fernández Mazuecos M, Vargas P. Genetically depauperate in the continent but tich in oceanic Islands: *Cistus monspeliensis* (Cistaceae) in the Canary Islands. PLoS One. 2011; 6. 10.1371/journal.pone.0017172 PMC3038934 21347265

[14] Sato JJ, Tasaka Y, Tasaka R, Gunji K, Yamamoto Y, Takada Y, et al. Effects of isolation by continental islands in the Seto Inland Sea, Japan, on genetic diversity of the large Japanese field mouse, *Apodemus speciosus* (Rodentia: Muridae), inferred from the mitochondrial *Dloop* region. Zoolog Sci. 2017; 34(2): 112–21, 10. doi: 10.2108/zs160113 28397602

[15] Hill R, Loxterman JL, Aho K. Insular biogeography and population genetics of dwarf mistletoe (*Arceuthobium americanum*) in the Central Rocky Mountains. Ecosphere. 2017; 8(5): e01810. doi: 10.1002/ecs2.1810

[16] Wang S, Zhu W, Gao X, Li X, Yan S, Liu X, et al. Population size and time since island isolation determine genetic diversity loss in insular frog populations. Mol Ecol. 2014; 23(3): 637–48. Epub 2013/12/20. doi: 10.1111/mec.12634 . 24351057

[17] Costanzi J-M, Steifetten Ø. Island biogeography theory explains the genetic diversity of a fragmented rock ptarmigan (*Lagopus muta*) population. Ecol Evol. 2019; 9(7): 3837–49. doi: 10.1002/ece3.5007 31015970 PMC6468070

[18] Yamada T, Maki M. Impact of geographical isolation on genetic differentiation in insular and mainland populations of Weigela coraeensis (Caprifoliaceae) on Honshu and the Izu Islands. J Biogeogr. 2012; 39(5): 901–17. 10.1111/j.1365-2699.2011.02634.x.

[19] García-Verdugo C, Sajeva M, La Mantia T, Harrouni C, Msanda F, Caujapé-Castells J. Do island plant populations really have lower genetic variation than mainland populations? Effects of selection and distribution range on genetic diversity estimates. Mol Ecol. 2015; 24(4): 726–41. doi: 10.1111/mec.13060 25580539

[20] Paulay G. Biodiversity on oceanic islands: its origin and extinction. Am Zool. 1994; 34(1): 134–44.

[21] Umino S. Geology of the Ogasawara Islands. Chizuchushin. 2008; 430: 6–7. (in Japanese).

[22] Umino S, Ishizuka O, Kanayama K. Geology of the Hahajima Retto District. Ibaraki, Japan: Geological Survey of Japan, AIST; 2016. (in Japanese with English abstract).

[23] Kaizuka S. Geology and geomorphology of the Bonin Islands. Bull Ogasawara Res. 1977; 1: 29–34. (in Japanese).

[24] Nakano S, Matsumoto A, Ohta Y, Nakamura H, Furukawa R, editors. K-Ar ages of volcanic rocks from Kito-Iwo-To and Minami-Iwo-To Islands. Japan Geoscience Union; 2009. (in Japanese with English abstract).

[25] Ohde S, Elderfield H. Strontium isotope stratigraphy of Kita-daito-jima Atoll, North Philippine Sea: implications for Neogene sea-level change and tectonic history. Earth and Planetary Science Letters. 1992; 113(4): 473–86. 10.1016/0012-821X(92)90125-F.

[26] Machida H, Ota Y, Kawana T, Moriwaki H, Nagaoka N, editors. Regional geomorphology of the Japanese Islands 7, Kyushu Island & Nansei Islands. Tokyo, Japan: Tokyo University Press; 2001. (in Japanese).

[27] Ohkawa T, Hayashi M. Trees and shrubs of the Ryukyu Islands. Tokyo, Japan: Bun-ichiSogo Shuppan; 2016. (in Japanese).

[28] Kamiya K. 300 million years history of the Ryukyu Islands described by stratum and fossils. Naha, Okinawa: Borderink; 2015. (in Japanese).

[29] Swenson U, Nylinder S, Munzinger J. Towards a natural classification of Sapotaceae subfamily Chrysophylloideae in Oceania and Southeast Asia based on nuclear sequence data. TAXON. 2013; 62(4): 746–70. doi: 10.12705/624.11

[30] Iwatsuki K, Yamazaki T, Boufford DE, Ohba H. Flora of Japan: Kodansha Tokyo; 1993.

[31] Toyoda T. Flora of Bonin Islands. Kanagawa, Japan: Aboc-sha; 2003. (in Japanese).

[32] Ohashi H, Kato H. SAPOTACEAE. In: Ohashi H, KAdota Y, Murata J, Yonekura K, Kihara H, editors. Wild flowers of Japan 4. Tokyo, Japan: Heibonsha; 2017. pp. 183. (in Japanese).

[33] Sugai K, Mori K, Murakami N, Kato H. Strong genetic structure revealed by microsatellite variation in *Callicarpa* species endemic to the Bonin (Ogasawara) Islands. J Plant Res. 2019; 132(6): 759–75. Epub 2019/10/19. doi: 10.1007/s10265-019-01144-4 . 31625126

[34] Ito M, Soejima A, Ono M. Genetic diversity of the endemic plants of the Bonin (Ogasawara) Islands. Evolution and speciation of island plants. Cambridge, UK: Cambridge University Press; 1998. pp. 141–54.

[35] Sugai K, Setsuko S, Nagamitsu T, Murakami N, Kato H, Yoshimaru H. Genetic differentiation in *Elaeocarpus photiniifolia* (Elaeocarpaceae) associated with geographic distribution and habitat variation in the Bonin (Ogasawara) Islands. J Plant Res. 2013; 126(6): 763–74. doi: 10.1007/s10265-013-0571-5 23748372

[36] Kato H, Tanishima A, Fujita T, Murakami N. Floral sexual expression of *Planchonella* in Japan. Ogasawara Research. 2016; 42: 9–21. (in Japanese).

[37] Abe T. Threatened pollination systems in native flora of the Ogasawara (Bonin) Islands. Annals of Botany. 2006; 98(2): 317–34. doi: 10.1093/aob/mcl117 16790463 PMC2803473

[38] Tsujimura M, Shimizu A, Karube H, Ohbayashi T, Murakami Y, Murakami N, et al. Disruption of local plant-pollinator ecosystems in the Ogasawara Islands by alien species. Ogasawara Research. 2016; 42: 23–64. (in Japanese).

[39] Pennington TD. Sapotaceae. In: Kubitzki K, editor. Flowering Plants · Dicotyledons: Celastrales, Oxalidales, Rosales, Cornales, Ericales. Heidelberg, Germany: Springer Berlin Heidelberg; 2004. pp. 390–421.

[40] Kawakami K, Mizusawa L, Higuchi H. Re-established mutualism in a seed-dispersal system consisting of native and introduced birds and plants on the Bonin Islands, Japan. Ecol Res. 2009; 24(4): 741–8. 10.1007/s11284-008-0543-8.

[41] Van Oosterhout C, Hutchinson WF, Wills DPM, Shipley P. MICRO-CHECKER: software for identifying and correcting genotyping errors in microsatellite data. Mol Ecol Notes. 2004; 4(3): 535–8. 10.1111/j.1471-8286.2004.00684.x.

[42] Goudet J. FSTAT (version 1.2): a computer program to calculate F-statistics. J Hered. 1995; 86(6): 485–6.

[43] El Mousadik A, Petit R. High level of genetic differentiation for allelic richness among populations of the argan tree [*Argania spinosa* (L.) Skeels] endemic to Morocco. Theoretical and applied genetics. 1996; 92(7): 832–9. doi: 10.1007/BF00221895 24166548

[44] Nei M. Molecular Evolutionary Genetics. New York, USA: Columbia University Press; 1987.

[45] Peakall R, Smouse PE. GENALEX 6: genetic analysis in Excel. Population genetic software for teaching and research. Mol Ecol Notes. 2006; 6(1): 288–95. 10.1093/bioinformatics/bts460 PMC3463245 22820204

[46] Nei M, Tajima F, Tateno Y. Accuracy of estimated phylogenetic trees from molecular data. J Mol Evol. 1983; 19(2): 153–70. 6571220 10.1007/BF02300753

[47] Bates D, Mächler M, Bolker B, Walker S. Fitting linear mixed-effects models using lme4. arXiv preprint arXiv:14065823. 2014.

[48] Hair JF, Ringle CM, Sarstedt M. PLS-SEM: Indeed a silver bullet. J Mark Theory Pract. 2011; 19(2): 139–52.

[49] Fox J, Weisberg S. An R companion to applied regression. Califolnia, USA: Sage publications; 2018.

[50] Akaike H, editor Information theory and an extension of the maximum likelihood principle. Proceedings of 2nd International Symposium on Information Theory; 1973; Budapest, Hungary: Springer Verlag.

[51] Burnham K, Anderson D. Model selection and multimodel inference: a practical information-theoretic approach. 2nd ed. New York, USA: Springer; 2002.

[52] R Core Team. R: a lunguage and environment for statistical computing. 2020.

[53] Falush D, Stephens M, Pritchard JK. Inference of population structure using multilocus genotype data: dominant markers and null alleles. Mol Ecol Notes. 2007; 7(4): 574–8. doi: 10.1111/j.1471-8286.2007.01758.x 18784791 PMC1974779

[54] Pritchard JK, Stephens M, Donnelly PJ. Inference of population structure using multilocus genotype data. Genetics. 2000; 155: 945–59. doi: 10.1093/genetics/155.2.945 10835412 PMC1461096

[55] Meirmans PG. Subsampling reveals that unbalanced sampling affects Structure results in a multi-species dataset. Heredity (Edinb). 2019; 122(3): 276–87. doi: 10.1038/s41437-018-0124-8 30026534 PMC6460757

[56] Evanno G, Regnaut S, Goudet J. Detecting the number of clusters of individuals using the software STRUCTURE: a simulation study. Mol Ecol. 2005; 14(8): 2611–20. doi: 10.1111/j.1365-294X.2005.02553.x 15969739

[57] Kopelman NM, Mayzel J, Jakobsson M, Rosenberg NA, Mayrose I. Clumpak: a program for identifying clustering modes and packaging population structure inferences across K. Mol Ecol Resour. 2015; 15(5): 1179–91. doi: 10.1111/1755-0998.12387 25684545 PMC4534335

[58] Langella O. POPULATIONS 1.2.32. http://wwwbioinformaticsorg/project/?group_id=842010.

[59] Wright S. Isolation by distance. Genetics. 1943; 28(2): 114–38. doi: 10.1093/genetics/28.2.114 17247074 PMC1209196

[60] Mantel N. The detection of disease clustering and a generalized regression approach. Cancer Res. 1967; 27: 209–20. 6018555

[61] Rousset F. Genetic differentiation and estimation of gene flow from *F*-statistics under isolation by distance. Genetics. 1997; 145: 1219–28. 9093870 10.1093/genetics/145.4.1219 PMC1207888

[62] Chen C, Lu RS, Zhu SS, Tamaki I, Qiu YX. Population structure and historical demography of *Dipteronia dyeriana* (Sapindaceae), an extremely narrow palaeoendemic plant from China: implications for conservation in a biodiversity hot spot. Heredity (Edinb). 2017; 119(2): 95–106. doi: 10.1038/hdy.2017.19 28379211 PMC5520545

[63] Maki M. Population genetics of threatened wild plants in Japan. J Plant Res. 2003; 116(2): 169–74. doi: 10.1007/s10265-003-0083-9 12736790

[64] Stuessy TF, Takayama K, López-Sepúlveda P, Crawford DJ. Interpretation of patterns of genetic variation in endemic plant species of oceanic islands. Bot J Linn Soc. 2014; 174(3): 276–88. doi: 10.1111/boj.12088 26074627 PMC4459035

[65] James JE, Lanfear R, Eyre-Walker A. Molecular evolutionary consequences of island colonization. Genome Biol Evol. 2016; 8(6): 1876–88. doi: 10.1093/gbe/evw120 27358424 PMC4943191

[66] Frankham R. Relationship of genetic variation to population dize in wildlife. Conserv Biol. 1996; 10(6): 1500–8. 10.1046/j.1523-1739.1996.10061500.x.

[67] Ryukyu Plant Research Group. Database of Ryukyu plants Japan: National Museum of Nature and Science; 2018 [cited 2021 4 March]. Available from: https://www.kahaku.go.jp/research/activities/project/hotspot_japan/ryukyus/db/. (in Japanese).

[68] Dunbar-Co S, Wieczorek AM, Morden CW. Molecular phylogeny and adaptive radiation of the endemic Hawaiian *Plantago* species (Plantaginaceae). Am J Bot. 2008; 95(9): 1177–88. doi: 10.3732/ajb.0800132 21632435

[69] López-Sepúlveda P, Takayama K, Greimler J, Crawford DJ, Penailillo P, Baeza M, et al. Progressive migration and anagenesis in *Drimys confertifolia* of the Juan Fernández Archipelago, Chile. J Plant Res. 2015; 128(1): 73–90. doi: 10.1007/s10265-014-0666-7 25292282 PMC4300435

[70] Setsuko S, Ohtani M, Sugai K, Nagamitsu T, Kato H, Yoshimaru H. Genetic variation of pantropical *Terminalia catappa* plants with sea-drifted seeds in the Bonin Islands: suggestions for transplantation guidelines. Plant Species Biol. 2017; 32(1): 13–24. 10.1111/1442-1984.12121.

[71] Takayama K, Ohi-Toma T, Kudoh H, Kato H. Origin and diversification of *Hibiscus glaber*, species endemic to the oceanic Bonin Islands, revealed by chloroplast DNA polymorphism. Mol Ecol. 2005; 14(4): 1059–71. 10.1111/j.1365-294X.2005.02462.x. 15773936

[72] Hayashi F, Shima A, Horikoshi K, Kawakami K, Segawa RD, Aotsuka T, et al. Limited overwater dispersal and genetic differentiation of the snake-eyed skink (*Cryptoblepharus nigropunctatus*) in the oceanic Ogasawara Islands, Japan. Zoolog Sci. 2009; 26(8): 543–9. doi: 10.2108/zsj.26.543 19719406

[73] Chiba S. Ecological diversity and speciation in land snails of the genus *Mandarina* from the Bonin Islands. Popul Ecol. 2002; 44(3): 179–87.

[74] Fukui A. Relationship between seed retention time in bird’s gut and fruit characteristics. Ornithol Sci. 2003; 2(1): 41–8.

[75] Tennekes H. The simple science of flight: from insects to jumbo jets. Massachusetts, USA: MIT press; 2009.

[76] Swenson U, Havran JC, Munzinger J, Mcloughlin S, Nylinder S. Metapopulation vicariance, age of island taxa and dispersal: A case study using the Pacific plant genus *Planchonella* (Sapotaceae). Syst Biol. 2019; 68(6): 1020–33. doi: 10.1093/sysbio/syz025 31157892 PMC6802573

[77] Liu U, Cossu TA, Dickie JB. Royal Botanic Gardens, Kew’s Seed Information Database (SID): A compilation of taxon-based biological seed characteristics or traits. Biodivers Inf Sci Stand. 2019; (1).

[78] Higgins SI, Nathan R, Cain ML. Are long-distance dispersal events in plants usually caused by nonstandard means of dispersal? Ecology. 2003; 84(8): 1945–56. 10.1890/01-0616.

[79] Heleno R, Vargas P. How do islands become green? Glob Ecol Biogeogr. 2015; 24(5): 518–26. 10.1111/geb.12273.

