# Supplementary material for: Contrasting genetic diversity between Planchonella obovata sensu lato (Sapotaceae) on old continental and young oceanic island populations in Japan
Source: PLoS One. 2022 Sep 2;17(9):e0273871. doi: 10.1371/journal.pone.0273871 (PMC9439235; doi:10.1371/journal.pone.0273871)
Supplement: S1 File — (DOCX) [file pone.0273871.s001.docx]

**Supporting Information**

**S1 Appendix. Details of nuclear microsatellite markers**

The total DNA of oftane *P. obovata* var. *obovata* plant from Hahajima in the Bonin Islands was used to develop nuclear SSR markers. A voucher specimen of the plant was deposited in the herbarium of the Forestry and Forest Products Research Institute (no. TF-K09-0051). DNA prepared for the NGS shotgun library was sequenced using a 454 GS Junior FLX Titanium platform (Roche, Basel, Switzerland), and 19,508 reads of at least 80 bp were obtained. The identification of SSR regions and design of primer pairs from the sequence data were performed using the program QDD 2.1 [1]. Microsatellite regions bordering sequences with more than five repeats of di- to hexanucleotide motifs and sequence lengths of 90–320 bp were selected. According to these criteria, 1,776 reads contained SSR loci. To eliminate redundancy, the similarity of sequences containing SSR regions was detected using all-against-all BLAST searching. Subsequently, 1,142 reads were selected from the whole set of sequences containing SSRs. PCR primer pairs were designed using Primer3 [2] implemented in QDD. Finally, 767 SSR primer pairs were designed. Amplification and polymorphism tests were performed for 48 selected primer pairs. We selected these primer pairs on the basis of their having single repeat motifs of di- and trinucleotides, with 9–22 repeats. All forward primers were fluorescently labeled at the 5′ end with one of four different tail sequences (A to D) shown in S1 Table, according to the method published by Blacket et al. [3]. All reverse primers were attached to a 5′-GTT-3′ sequence at the 5′-end of the sequence, to reduce stuttering due to the addition of non-templated adenine base pairs by Taq DNA polymerase [4]. PCR amplification was conducted using QIAGEN Multiplex PCR Kits (QIAGEN). Multiplex PCR was performed for each of the four primer pair sets using the following program: initial denaturation at 95ºC for 15 min, followed by 35 cycles of denaturation at 95ºC for 30 s, annealing at 56ºC for 90 s, and extension at 72ºC for 60 s, and a final extension step was performed at 60ºC for 30 min. The PCR products were submitted for genotyping on an ABI3130 sequencer (Applied Biosystems, Waltham, MA, USA) using the GeneScan 600 LIZ Size Standard (Applied Biosystems). The genotypes were scored using GeneMapper 4.0 (Applied Biosystems). To evaluate polymorphisms in these markers, a total of 24 *Planchonella* samples were used, consisting of 20 *P. obovata* *s.l*. and four *P. boninensis* to check cross species amplification. Eighteen out of 48 primer pairs showed clear amplification (S1 Table).

**S2 Appendix. Details of ABC analysis**

Bayesian clustering analysis detected clear genetic structure among three island groups: the Bonin Islands; the Volcano Islands; and the Yaeyama and Daito Islands (see details in Results). In order to estimate the population demography of these three island groups, we conducted approximate Bayesian computation (ABC). We used the same data set as was used for the STRUCTURE analysis: 122, 35, and 84 individuals for the Bonin, Volcano, and Yaeyama + Daito groups, respectively. As there are various patterns in combinations of population demography—population size change, population divergence, and migration patterns—we sequentially executed ABC analyses [5, 6]. In the first step, we applied single population size change models for each island group (Fig 2a). In the second step, we applied three-population divergence models without migration for the three island groups (Fig 2b). Finally, in the third step, we examined divergence models with and without migration (Fig 2c). For the first step, we used six summary statistics: the averages and standard deviations of the number of alleles (*A*), gene diversity (*H*_E_), and allele size range (*R*) for 11 loci overall. For the second and third steps, we used 25 summary statistics, of which 18 summary statistics (6 × 3 island groups) were the same as the first step (S6 Fig). We also used average *A*, *H*_E_, and *R* for overall groups, and pairwise *F*_ST_ and overall *F*_ST_ among the three groups for a total of 11 loci. Arlsumstat version 3.5.2 was used for calculation of the summary statistics [7].

Three single population size change models—the standard neutral model (SNM), a population growth model (PGM), and a size reduction model (SRM)—were used (Fig 2a). The SNM assumes that a population maintains its current effective population size (*N*_CUR_) over time, and has one structural parameter, *N*_CUR_. The PGM assumes that the population size has expanded exponentially from the past to the present, with the growth rate *G*; *N*_T_ = *N*_CUR_ exp (*G* × *T*), *N*_T_ is the effective population size at time *T*, and the unit of all time parameters is generations ago. The PGM has two structural parameters, *N*_CUR_ and *G*. The SRM assumes that population size has shrunk at time *T*_1_ from an ancestral population size *N*_ANC_; *N*_ANC_ = *N*_CUR_ × *RN*_ANC_, *RN*_ANC_ is a relative ancestral effective population size for *N*_CUR_. The SRM has three structural parameters, *N*_CUR_, *RN*_ANC_, and *T*_1_. The prior distributions of the parameters are summarized in S7 Table. For the mutation model of microsatellites, a generalized stepwise mutation model (GSM) was assumed [8]. The GSM has two parameters, mutation rate (*μ*) and geometric parameter (*P*_GSM_). *P*_GSM_ ranges from 0 to 1, and represents the proportion of mutations that change allele sizes by more than one step. A value of zero means a strict stepwise mutation model. The value of 1 × 10^-4^ was used for the mean value of *μ* among 11 loci [6]. The prior distribution for the value of *μ* for each locus was randomly drawn from the gamma distribution with shape and rate parameters. The prior distribution of the shape parameter was drawn from a uniform distribution from 0.5 to 5, and the rate parameter was calculated by shape / mean value of *μ*. The prior distribution of the mean value of *P*_GSM_ among 11 loci was drawn from a uniform distribution from 0 to 1, and each locus value was randomly drawn from a beta distribution with parameters *a* and *b*. The values of *a* and *b* were calculated as 0.5 + 199 × mean value of *P*_GSM_ and a × (1 – mean value of *P*_GSM_) / mean value of *P*_GSM_, respectively, as described by Excoffier et al. [9].

Prior distributions were generated using R version 4.0.2 [10]. Three population size change models were simulated 10,000 times each using fastsimcoal2 version 2.6.0.3 [11], and summary statistics were calculated using arlsumstat. Three models were compared using the ABC random forest (ABC-RF) approach implemented in the abcrf package version 1.8.1 of R [12]. The number of trees in the random forest was set to 1,000. The classification error and posterior probability of the best model was calculated. For the best model selected by ABC-RF, 2 × 10^5^ simulations were repeated, and summary statistics were calculated. With 1,000 simulations nearest the observed data set, the posterior distributions of parameters were estimated using neural network regression, as implemented in the abc package version 2.1 of R [13, 14]. The number of neural networks was set to 20. Logit transformation was used to keep the estimated value within the prior range. The posterior mode was estimated using the density function of R. The 95% highest posterior density (HPD) was estimated using the coda package version 0.19.3 [15].

Five three-population divergence models, Models1–5, were applied (Fig 2b). As in the single population size change, this analysis showed that the SNM was selected in both the Bonin and Yaeyama + Daito groups, and the posterior distributions of *N*_CUR_ in the two groups were almost the same (see detail in Results). We set the common current effective population size parameter (*N*_BYD_) for the Bonin and Yaeyama + Daito groups. For the current effective population size of the Volcano group, *N*_V_ was set. All five models had distinct ancestral effective population size parameter values (*N*_ANC_). Model1 assumes that three populations simultaneously diverged at time *T*_1_, and has four structural parameters. Model2 assumes that initially, the Volcano group diverged from the ancestral population at time *T*_2_, and then the Bonin and Yaeyama + Daito groups diverged, and has five structural parameters. Model3 and Model4 assume that initially the Bonin and Yaeyama + Daito groups diverged at time *T*_2_, and then the Volcano group diverged from the Bonin or Yaeyama + Daito groups at time *T*_1_, and has five structural parameters. Model5 assumes that initially the Bonin and Yaeyama + Daito groups diverged at time *T*_2_ and then the Volcano group was created by admixture between the Bonin and Yaeyama + Daito groups with admixture rates *P*_B_ and 1 – *P*_B_, respectively, at time *T*_1_, and has six structural parameters. The prior distributions of the parameters are summarized in S7 Table. The mutation model used in the single population size change analysis was used. Model comparison was conducted as for the single population size change analysis. For the best model, 1 × 10^6^ simulations were run, and summary statistics were calculated. Parameter estimation was also conducted as in single population size change analysis.

Using the best model selected in the three-population divergence analysis (Model3; see details in Results), we compared four models: three with migration and one without migration. Three migration patterns were proposed (Fig 2c). Pattern M1 assumes that migration exists between all three island group pairs. Pattern M2 assumes that migration exists only between the Bonin and Volcano groups, because the distance between these groups is the shortest among the three group pairs. Pattern M3 assumes that migration exists only between the Bonin and Yaeyama + Daito groups, because the pairwise *F*_ST_ value is the smallest among the three group pairs (S6 Fig). The unit of migration was a number of migrants per generation (*Nm*). The prior distribution of *Nm* was drawn from a uniform distribution from 1 to 10. When running simulations, *Nm* was divided by the effective population size of the source population toward the coalescence, and then the calculated migration rate was passed to the coalescent simulator. Model comparison was conducted as for single population size change analysis.

To assess the fit of the model to the observed data, posterior predictive simulation with 1,000 randomly drawn posterior samples was conducted [16]. Summary statistics were calculated and compared to the observed data.

**References for S1 and S2 Appendix**

1. Meglécz E, Costedoat C, Dubut V, Gilles A, Malausa T, Pech N, et al. QDD: a user-friendly program to select microsatellite markers and design primers from large sequencing projects. Bioinformatics. 2010; 26(3): 403-4.

2. Rozen S, Skaletsky H. Primer3 on the WWW for general users and for biologist programmers. Bioinformatics methods and protocols: Springer; 2000. pp. 365-86.

3. Blacket M, Robin C, Good R, Lee S, Miller A. Universal primers for fluorescent labelling of PCR fragments — an efficient and cost-effective approach to genotyping by fluorescence. Mol Ecol Resour. 2012; 12(3): 456-63.

4. Brownstein MJ, Carpten JD, Smith JR. Modulation of non-templated nucleotide addition by Taq DNA polymerase: primer modifications that facilitate genotyping. Biotechniques. 1996; 20(6): 1004-6, 8-10. Epub 1996/06/01. PubMed PMID: 8780871.

5. Chen C, Lu RS, Zhu SS, Tamaki I, Qiu YX. Population structure and historical demography of *Dipteronia dyeriana* (Sapindaceae), an extremely narrow palaeoendemic plant from China: implications for conservation in a biodiversity hot spot. Heredity (Edinb). 2017; 119(2): 95-106. doi: 10.1038/hdy.2017.19.

6. Setsuko S, Sugai K, Tamaki I, Takayama K, Kato H, Yoshimaru H. Genetic diversity, structure, and demography of *Pandanus boninensis* (Pandanaceae) with sea drifted seeds, endemic to the Ogasawara Islands of Japan: Comparison between young and old islands. Mol Ecol. 2020; 29(6): 1050-68.

7. Excoffier L, Lischer HE. Arlequin suite ver 3.5: a new series of programs to perform population genetics analyses under Linux and Windows. Mol Ecol Resour. 2010; 10(3): 564-7. Epub 2011/05/14. doi: 10.1111/j.1755-0998.2010.02847.x. PubMed PMID: 21565059.

8. Estoup A, Jarne P, Cornuet JM. Homoplasy and mutation model at microsatellite loci and their consequences for population genetics analysis. Mol Ecol. 2002; 11(9): 1591-604. Epub 2002/09/05. PubMed PMID: 12207711.

9. Excoffier L, Laval G, Schneider S. Arlequin (version 3.0): an integrated software package for population genetics data analysis. Evol Bioinform Online. 2005; 1: 47.

10. R Core Team. R: a lunguage and environment for statistical computing. 2020.

11. Excoffier L, Foll M. fastsimcoal: a continuous-time coalescent simulator of genomic diversity under arbitrarily complex evolutionary scenarios. Bioinformatics. 2011; 27(9): 1332-4. Epub 2011/03/15. doi: 10.1093/bioinformatics/btr124. PubMed PMID: 21398675.

12. Pudlo P, Marin JM, Estoup A, Cornuet JM, Gautier M, Robert CP. Reliable ABC model choice via random forests. Bioinformatics. 2016; 32(6): 859-66. Epub 2015/11/22. doi: 10.1093/bioinformatics/btv684. PubMed PMID: 26589278.

13. Blum MGB, Francois O. Non-linear regression models for Approximate Bayesian Computation. Statistics and Computing. 2010; 20: 63-73.

14. Csilléry K, François O, Blum MGB. abc: an R package for approximate Bayesian computation (ABC). Methods Ecol Evol. 2012; 3(3): 475-9. doi: <https://doi.org/10.1111/j.2041-210X.2011.00179.x>.

15. Plummer M, Best N, Cowles K, Vines K. CODA: Convergence diagnosis and output analysis for MCMC. R News, 6, 7-11. URL: <http://CRAN> R-project org/doc/Rnews. 2006.

16. Gelman A, Carlin JB, Stern HS, Dunson DB, Vehtari A, Rubin DB. Bayesian data analysis. 3rd ed. Florida, USA: CRC Press; 2014.

**S1 Table.** Characteristics of 18 SSR primers designed for *Planchonella obovata* *s.l*.

| Locus name | Accession no. |  | Primer squence (5'-3')^a^ | Repeat | Multiplex panel | *P. obovata s.l*. (*N* =20) | | | | | *P. boninensis* (*N* = 4)^b^ |
| --- | --- | --- | --- | --- | --- | --- | --- | --- | --- | --- | --- |
|  |  |  |  |  |  | Allele size range | *A* | *H*_O_ | *H*_E_ | *F*_IS_ |  |
| Po016 | LC076460 | F: | [Tail C] CTAAACGGTGCCTAAATTGAGTGT | (AT)9 | 2 | 149-157 | 5 | 0.42 | 0.67 | 0.37 | No |
|  |  | R: | gttCATTAATGGAATTGGAATTTGTCC |  |  |  |  |  |  |  |  |
| Po017 | LC076464 | F: | [Tail D] GGGTTTAAGCTGGAATTCTCTCTG | (AG)11 | 2 | 116-122 | 3 | 0.4 | 0.59 | 0.32 | No |
|  |  | R: | gttGTGTGCATGAAAGGAAGGAAA |  |  |  |  |  |  |  |  |
| Po116 | LC076449 | F: | [Tail A] CATTGGATATGTAAATGTAGATGATGG | (AT)9 | 1 | 100-110 | 6 | 0.58 | 0.79 | 0.26 | No |
|  |  | R: | gttCAGTGTGGTTGCTAGATAGGGTTT |  |  |  |  |  |  |  |  |
| Po124 | LC076456 | F: | [Tail B] TGAACAACGGCCTTCTTAATCATA | (AG)14 | 1 | 102-120 | 4 | 0.2 | 0.67 | 0.7 | 1 |
|  |  | R: | gttCCCAACTAGTTTGTCCAGAGGTAA |  |  |  |  |  |  |  |  |
| Po182 | LC076458 | F: | [Tail A] GACTTAGGACACGATACAAGGTGG | (AG)9 | 2 | 261-285 | 6 | 0.75 | 0.76 | 0.02 | 4 |
|  |  | R: | gttATTCTCTTAGCTATTGACGCGTTT |  |  |  |  |  |  |  |  |
| Po188 | LC076457 | F: | [Tail B] GATTTGAATGGCCTAGACTCCTCT | (AG)9 | 1 | 174-203 | 8 | 0.55 | 0.63 | 0.12 | 2 |
|  |  | R: | gttTTTCTTGTGGAAGGAGACAAGATT |  |  |  |  |  |  |  |  |
| Po200 | LC076453 | F: | [Tail C] AAACACCCTGAATAAATGCTTACG | (AG)11 | 1 | 188-205 | 4 | 0.19 | 0.37 | 0.49 | 1 |
|  |  | R: | gttTCAACTGTCTTTACTATGAGCGATG |  |  |  |  |  |  |  |  |
| Po267 | LC076462 | F: | [Tail D] TATGGGACTGGTTTGCCTATTAAG | (AT)9 | 2 | 336-346 | 5 | 0.65 | 0.67 | 0.03 | No |
|  |  | R: | gttTTACTCAACTGTGATCCTGCAGTC |  |  |  |  |  |  |  |  |
| Po281 | LC076455 | F: | [Tail D] AAATGGAAAGCACAAATGTCATAA | (AT)10 | 1 | 114-134 | 6 | 0.58 | 0.77 | 0.25 | 1 |
|  |  | R: | gttTAATTGACAATGACCCAACGAAAT |  |  |  |  |  |  |  |  |
| Po290 | LC076463 | F: | [Tail D] TGGTCATAATGAAAGGATACGTCA | (AT)9 | 2 | 223-233 | 6 | 0.6 | 0.79 | 0.24 | No |
|  |  | R: | gttTCATAGACAGAGGATTCCAGTGGT |  |  |  |  |  |  |  |  |
| Po352 | LC076465 | F: | [Tail B] CACAATTGATTGGAGAATGAGAAA | (AT)9 | 2 | 212-216 | 3 | 0.2 | 0.19 | -0.08 | 7 |
|  |  | R: | gttTCGATGAGATATTATCAAGGCACA |  |  |  |  |  |  |  |  |
| Po419 | LC076461 | F: | [Tail C] GGTCATTAGAAGTCAGTGAATCTGG | (ACC)9 | 2 | 208-224 | 4 | 0.5 | 0.47 | -0.06 | 1 |
|  |  | R: | gttATAATCACAATTGGGCATACCTTG |  |  |  |  |  |  |  |  |
| Po506 | LC076450 | F: | [Tail A] CCACCATCGTCTAAGATTGAGATT | (AAT)15 | 1 | 185-236 | 13 | 0.8 | 0.86 | 0.07 | 4 |
|  |  | R: | gttGTAGAAGTTGGGAGGAATAGGGTG |  |  |  |  |  |  |  |  |
| Po564 | LC076466 | F: | [Tail B] CTAACAGAGAATTGGAAACCAAGG | (AG)15 | 2 | 124-146 | 12 | 0.7 | 0.83 | 0.15 | 1 |
|  |  | R: | gttGTTCGAGTAGGAATCATCCATCA |  |  |  |  |  |  |  |  |
| Po579 | LC076454 | F: | [Tail D] TGGCAGTGTTTATGTCATGGTATC | (AG)11 | 1 | 294-304 | 6 | 0.3 | 0.49 | 0.39 | No |
|  |  | R: | gttGGAAATTGAAACTGAAGAAAGGAA |  |  |  |  |  |  |  |  |
| Po583 | LC076452 | F: | [Tail C] GGAGATAGTTCTATGTTTGGTCATGC | (AAG)12 | 1 | 86-107 | 6 | 0.25 | 0.81 | 0.69 | 1 |
|  |  | R: | gttGGACGAAGAAGAAGAAGCAGTAGT |  |  |  |  |  |  |  |  |
| Po587 | LC076459 | F: | [Tail A] AGGGTTGGGTACTTGAGGTTACTT | (AC)11 | 2 | 183-190 | 4 | 0.6 | 0.59 | -0.02 | 1 |
|  |  | R: | gttATCAATGATATGACAAGCCACAAA |  |  |  |  |  |  |  |  |
| Po623 | LC076451 | F: | [Tail C] AAGAATACATTAATCGGTGGGACA | (AG)12 | 1 | 241-259 | 5 | 0.42 | 0.65 | 0.35 | No |
|  |  | R: | gttCTAGCCATGTTAGCTACCCAGC |  |  |  |  |  |  |  |  |

*A*; number of alleles, *H*_O_; observed heterozygosity, *H*_E_; gene diversity, *F*_IS_; fixation index

^a^ Tails of the forward primers are indicted as follows: [Tail A] = 5'-GCCTCCCTCGCGCCA-3'; [Tail B] = 5’-GCCTTGCCAGCCCGC-3’; [Tail C] = 5’-CAGGACCAGGCTACCGTG-3’; and [Tail D] = 5’-CGGAGAGCCGAGAGGTG-3’. Reverse primer sequences contained the PIG-tail sequence, gtt.

^b^ Transferability of the 18 SSR markers for *P. boninensis*. The number of alleles is given for loci for which amplification was successful.

**S2 Table.** The three best models explaining the genetic diversity of all *Planchonella obovata* *s.l*. populations, selected according to Akaike’s information criterion (AIC). The best three models, including null models, are shown.

| *Data set* | *model rank* | *A*_R_ |  |  | *H*_E_ |  |
| --- | --- | --- | --- | --- | --- | --- |
|  |  | *selected model* | *ΔAIC* |  | *model* | *ΔAIC* |
| All | 1 | *c+origin* | 0.0 |  | *c+origin* | 0.0 |
| populations | 2 | *c+area+origin* | 4.8 |  | *c* | 9.4 |
|  | 3 | *c+area* | 10.0 |  | *c+area+dist* | 9.9 |
|  |  |  |  |  |  |  |
| Oceanic | 1 | *c+age* | 0.0 |  | *c* | 0.0 |
| populations | 2 | *c+age+area* | 6.0 |  | *c+age* | 1.9 |
|  | 3 | *c+age+dist* | 12.4 |  | *c+area* | 10.0 |

*A*_R_, allelic richness; *H*_E_, gene diversity; *ΔAIC*, the difference in AIC between the model considered and the most parsimonious one; *c*, intercept; *area*, island area, *dist*, distance to nearest continent; *origin*, oceanic (1) or continental (0) islands; *age*, island age (0: young, 1: middle).

**S3 Table.** Explanatory variables of the linear mixed-effect models (random effect: locus) that best explained the level of allelic richness and gene diversity of the all *Planchonella obovata s.l*.

| *Data set* | *Response variables* | *model rank* | *Explanatory variables* | *Regression coefficients* | *s.e.* | *t* |
| --- | --- | --- | --- | --- | --- | --- |
|  |  |  |  |  |  |  |
| All | *A*_R_ | 1 | *c* | 5.49 | 0.53 | 10.44 |
| populations |  |  | *origin* | -0.99 | 0.23 | -4.37 |
|  | *H*_E_ | 1 | *c* | 0.76 | 0.07 | 11.37 |
|  |  |  | *origin* | -0.13 | 0.03 | -4.10 |
|  |  |  |  |  |  |  |
| Oceanic | *A*_R_ | 1 | *c* | 3.17 | 0.57 | 5.59 |
| populations |  |  | *age* | 1.38 | 0.29 | 4.67 |
|  | *H*_E_ | 1 | *c* | 0.63 | 0.06 | 9.95 |
|  |  | 2 | *c* | 0.54 | 0.08 | 7.18 |
|  |  |  | *age* | 0.09 | 0.04 | 2.14 |

Abbreviations: *A*_R_, allelic richness; *H*_E_, gene diversity; *c*, intercept; *origin*, oceanic (1) or continental (0) islands; *age*, island age (0: young, 1: middle)

**S4 Table.** Proportion of votes by random forest, posterior probability of the best model, classification error rate and posterior mode and 95% highest posterior density (HPD) of parameters of the best model in single population size change analysis.

| Island | Proportion of votes ^a^ | | | Posterior | Classification | Parameter (posterior mode / 95%HPD) | | | | |
| --- | --- | --- | --- | --- | --- | --- | --- | --- | --- | --- |
| group | SNM | PGM | SRM | probability | error rate | *N*_CUR_ | *RN*_ANC_ | *T*_1_ | *shape* | mean *P*_GSM_ |
| Bonin | **0.588** | 0.389 | 0.023 | 0.757 | 0.279 | 10436 |  |  | 1.96 | 0.418 |
|  |  |  |  |  |  | (6517–18411) |  |  | (0.73–4.76) | (0.243–0.514) |
| Volcano | 0.070 | 0.166 | **0.764** | 0.791 | 0.255 | 1829 | 26.1 | 4703 | 1.45 | 0.150 |
|  |  |  |  |  |  | (401–4561) | (10.0–732.8) | (769–71100) | (0.60–4.63) | (0.000–0.534) |
| Yaeyama + Daito | **0.696** | 0.292 | 0.012 | 0.789 | 0.268 | 12468 |  |  | 2.32 | 0.454 |
|  |  |  |  |  |  | (7283–23472) |  |  | (0.70–4.66) | (0.286–0.559) |

^a^ Best model was shown in bold.

**S5 Table.** Confusion matrices and classification error rates estimated by random forest (RF).

|  |  | Proportion of models predicted by RF ^a^ | | | | | Classification |
| --- | --- | --- | --- | --- | --- | --- | --- |
| Compared model set | True model | Model1 | Model2 | Model3 | Model4 | Model5 | error rate |
| Five divergence models | Model1 | **0.633** | 0.154 | 0.070 | 0.073 | 0.070 | 0.367 |
|  | Model2 | 0.248 | **0.702** | 0.020 | 0.020 | 0.011 | 0.298 |
|  | Model3 | 0.200 | 0.033 | **0.686** | 0.015 | 0.066 | 0.314 |
|  | Model4 | 0.200 | 0.031 | 0.017 | **0.684** | 0.068 | 0.316 |
|  | Model5 | 0.225 | 0.033 | 0.196 | 0.192 | **0.354** | 0.646 |
|  |  |  |  |  |  |  |  |
|  |  | Model1 | Model2 | Model3 | Model4 |  |  |
| Four divergence models | Model1 | **0.663** | 0.154 | 0.091 | 0.092 |  | 0.337 |
|  | Model2 | 0.255 | **0.698** | 0.023 | 0.024 |  | 0.302 |
|  | Model3 | 0.217 | 0.033 | **0.729** | 0.020 |  | 0.271 |
|  | Model4 | 0.214 | 0.032 | 0.024 | **0.730** |  | 0.270 |
|  |  |  |  |  |  |  |  |
|  |  | Model1 | Model3 |  |  |  |  |
| Two divergence models | Model1 | **0.905** | 0.095 |  |  |  | 0.095 |
|  | Model3 | 0.274 | **0.726** |  |  |  | 0.274 |
|  |  |  |  |  |  |  |  |
|  |  | Model3M1 | Model3M2 | Model3M3 | Model3 |  |  |
| With/without migration models | Model3M1 | **0.931** | 0.028 | 0.038 | 0.003 |  | 0.069 |
|  | Model3M2 | 0.082 | **0.789** | 0.070 | 0.059 |  | 0.211 |
|  | Model3M3 | 0.098 | 0.039 | **0.826** | 0.036 |  | 0.174 |
|  | Model3 | 0.047 | 0.215 | 0.148 | **0.590** |  | 0.410 |

^a^ Proportion of models correctly classified was shown in bold.

**S6 Table.** Posterior mode and 95% highest posterior density (HPD) of parameters in Model3, which was the best model selected by ABC-RF.

|  | Posterior | 95% HPD | |
| --- | --- | --- | --- |
| Parameter | mode | Lower | Upper |
| *N*_BYD_ | 15213 | 8743 | 29273 |
| *N*_V_ | 1710 | 690 | 4537 |
| *N*_ANC_ | 3789 | 126 | 24022 |
| *T*_1_ | 1623 | 295 | 5169 |
| *T*_2_ | 8652 | 2888 | 21509 |
| *shape* | 2.61 | 1.02 | 4.63 |
| mean *P*_GSM_ | 0.486 | 0.328 | 0.573 |

**S7 Table.** Prior distribution for population demographic models.

| Analysis | Parameter | Model | Distribution |
| --- | --- | --- | --- |
| Single population size change | *N*_CUR_ | SNM, PGM | Uniform (10, 5×10^4^) |
|  | *N*_CUR_ | SRM | Uniform (10, 5×10^3^) |
|  | *G* | PGM | Uniform (-0.001, 0) |
|  | *RN*_ANC_ | SRM | Log-uniform (10, 10^3^) |
|  | *T*_1_ | SRM | Log-uniform (10^2^, 10^5^) |
|  | *shape* | All models | Uniform (0.5, 5) |
|  | mean *P*_GSM_ | All models | Uniform (0, 1) |
|  |  |  |  |
| Three-population divergence and migration pattern | *N*_BYD_ | All models | Uniform (10, 5×10^4^) |
|  | *N*_V_ | All models | Uniform (10, 5×10^3^) |
|  | *N*_ANC_ | All models | Uniform (10, 1×10^5^) |
|  | *T*_1_ | All models | Log-uniform (10^2^, 10^5^) |
|  | *T*_2_ ^a^ | Model2–5 | Log-uniform (10^2^, 10^5^) |
|  | *P*_B_ | Model5 | Uniform (0, 1) |
|  | *Nm* | Model3M1–3 | Uniform (1, 10) |
|  | *shape* | All models | Uniform (0.5, 5) |
|  | mean *P*_GSM_ | All models | Uniform (0, 1) |

^a^ Always *T*_1_ < *T*_2_.


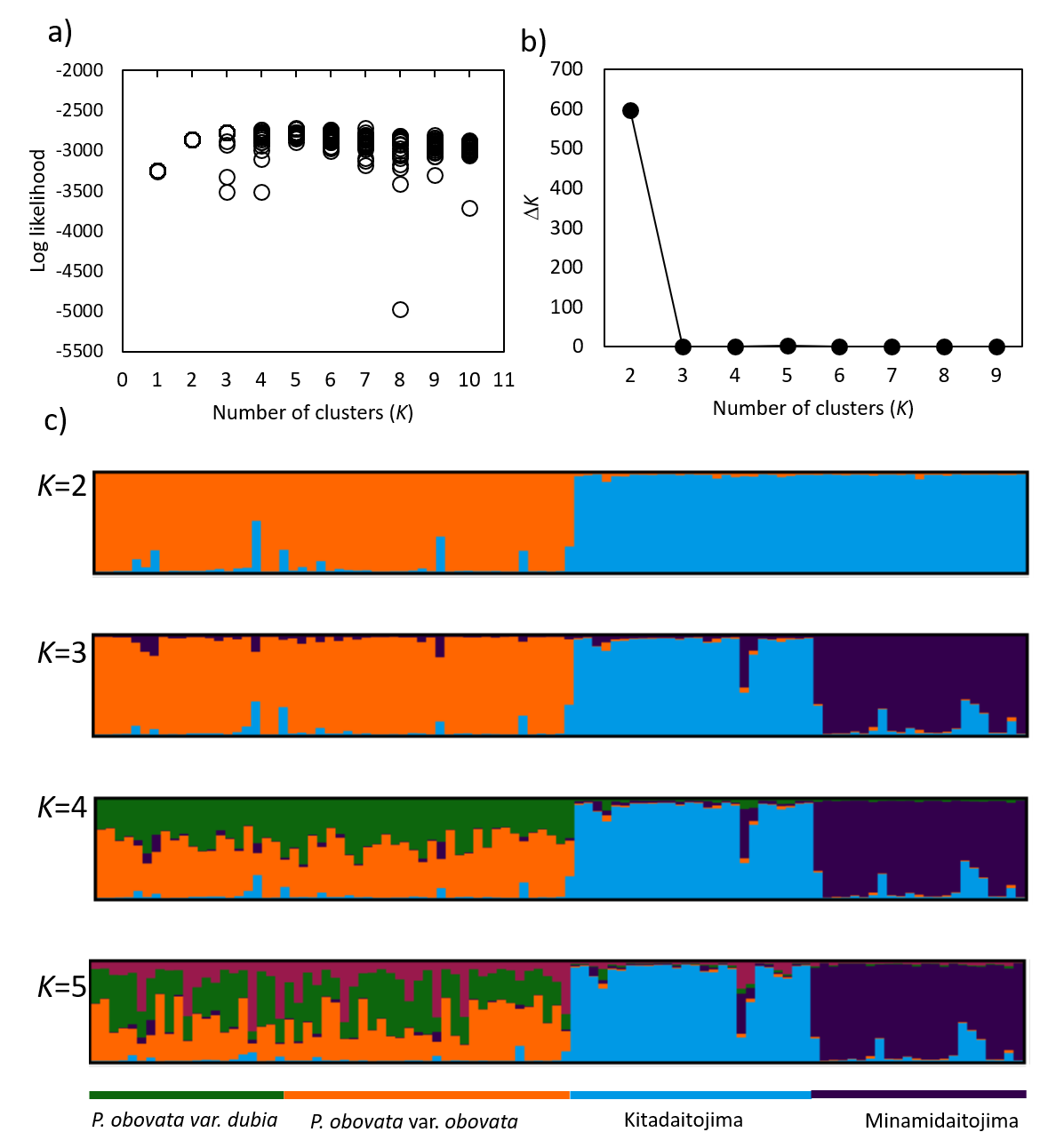


**S1 Fig.** Results of STRUCTURE for *Planchonella obovata* var. *dubia* (CC4_dubia, *n* = 17), *P. obovata* var. *obovata* (CC4, *n* = 35) on Chichijima island, and *P. obovata* *s.l*. on Kitadatitojima (*n* = 26) and Minamidaitojima Islands (*n* = 23). (a) Changes in loglikelihood and (b) Δ*K* as the number of clusters (*K* ranging from 1 to 10), (c) barplots of 101 genotypes at *K* = 2 to 5. Vertical columns represent individuals; heights of bars are proportional to the posterior means of the estimated admixture proportions.

**
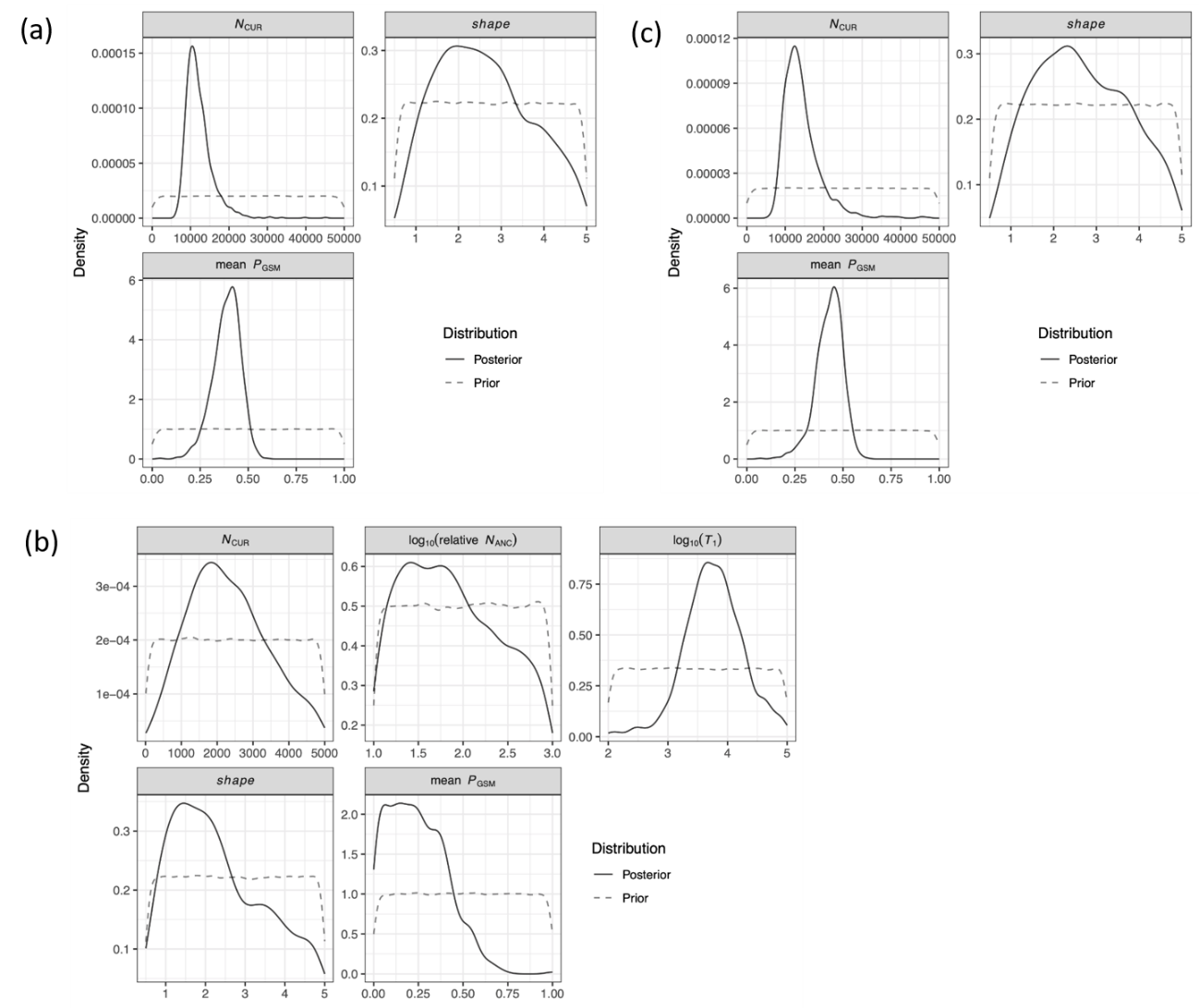
**

**S2 Fig.** Posterior and prior distributions of standard neutral model (SNM) and size reduction model (SRM). SNM for Bonin, SRM for Volcano and SNM for Yaeyama + Daito groups (a, b and c, respectively).


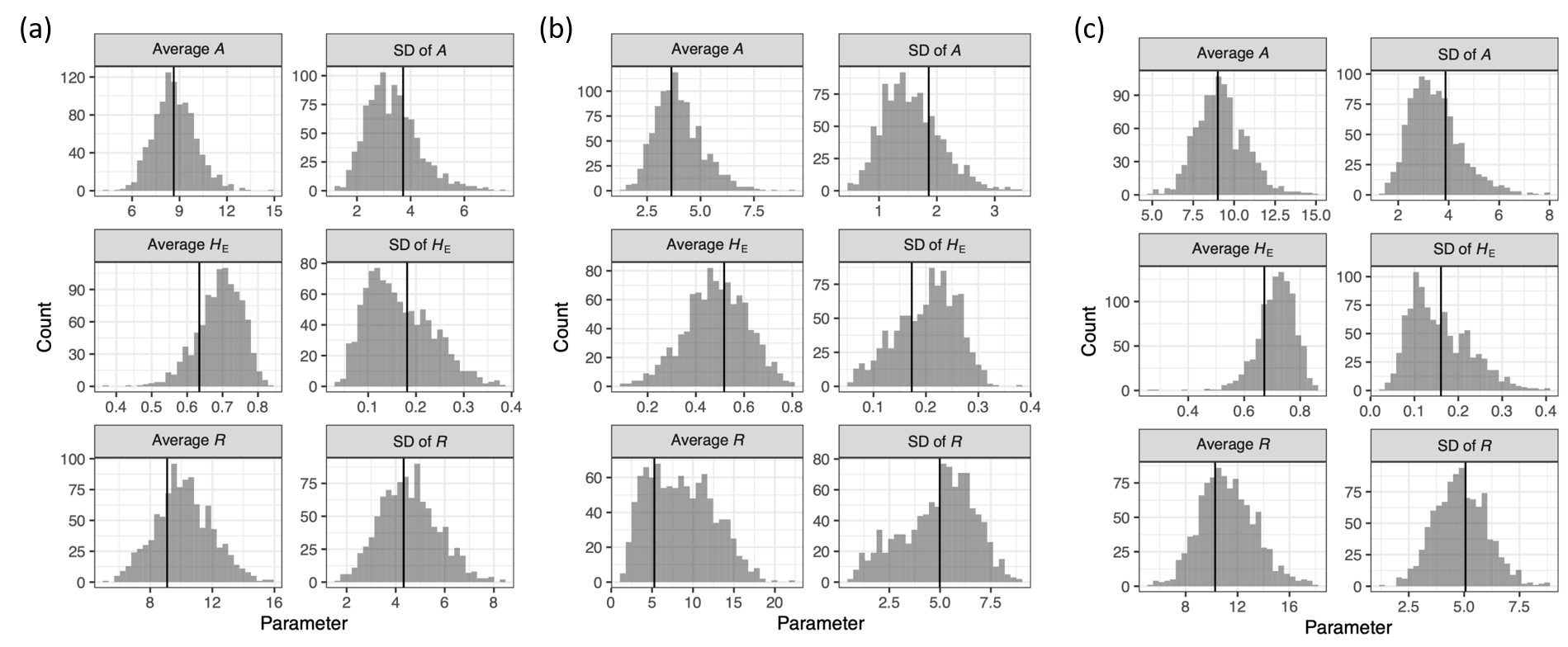


**S3 Fig.** Predicted and observed values (histogram and vertical bar, respectively) for Bonin, Volcano and Yaeyama + Daito groups (a, b and c, respectively). Posterior predictive simulation was performed using the best model. *A*, number of alleles; *H*_E_, gene diversity; *R*, allele size range; SD, standard deviation.


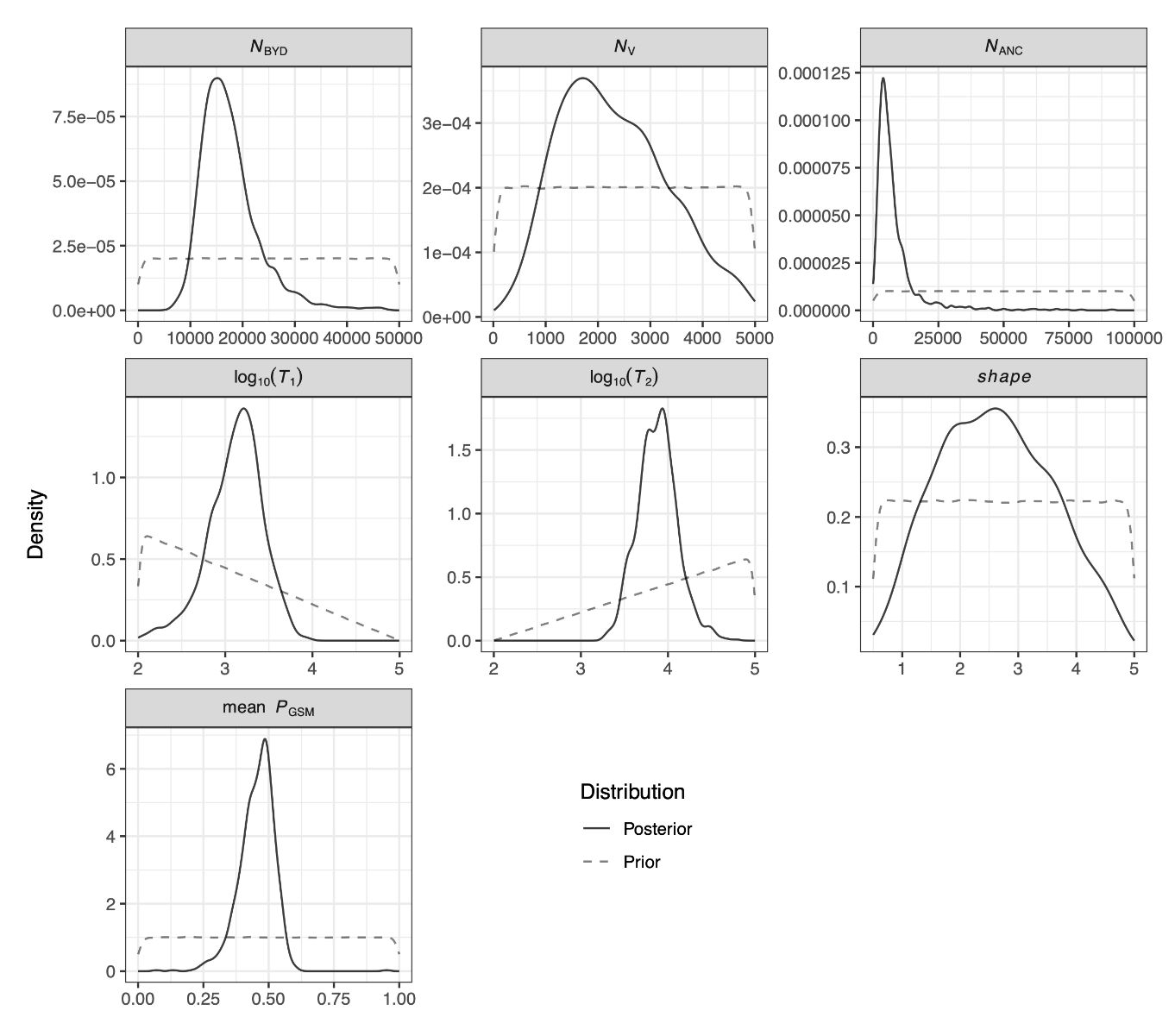


**S4 Fig.** Posterior and prior distributions of three-population divergence model (Model3).
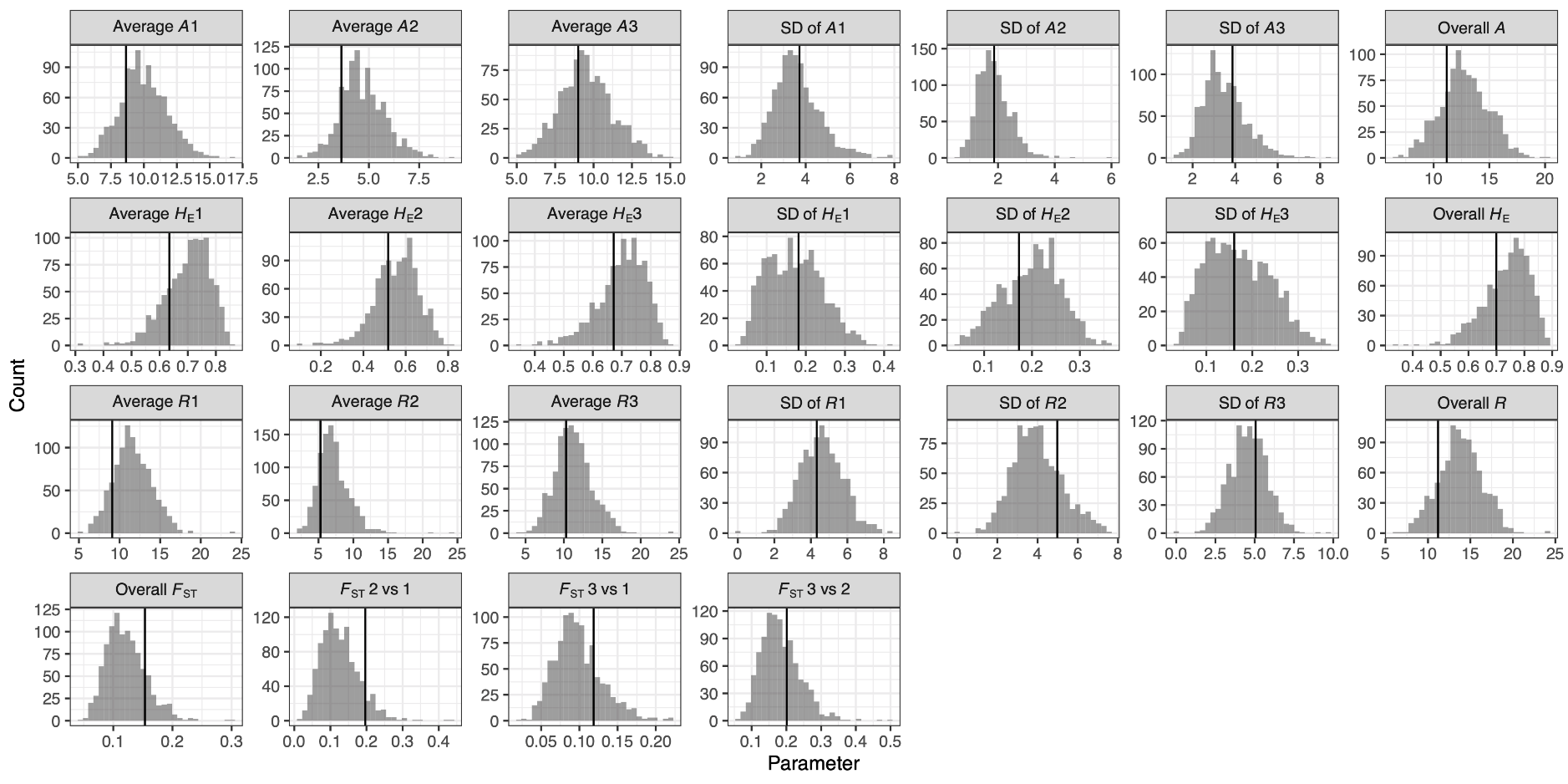


**S5 Fig.** Predicted and observed values (histogram and vertical bar, respectively) in three-population divergence analysis. Posterior predictive simulation was performed using the best model (Model3). *A*, number of alleles; *H*_E_, gene diversity; *R*, allele size range; SD, standard deviation. 1, 2 and 3 indicate Bonin, Volcano and Yaeyama + Daito groups, respectively.


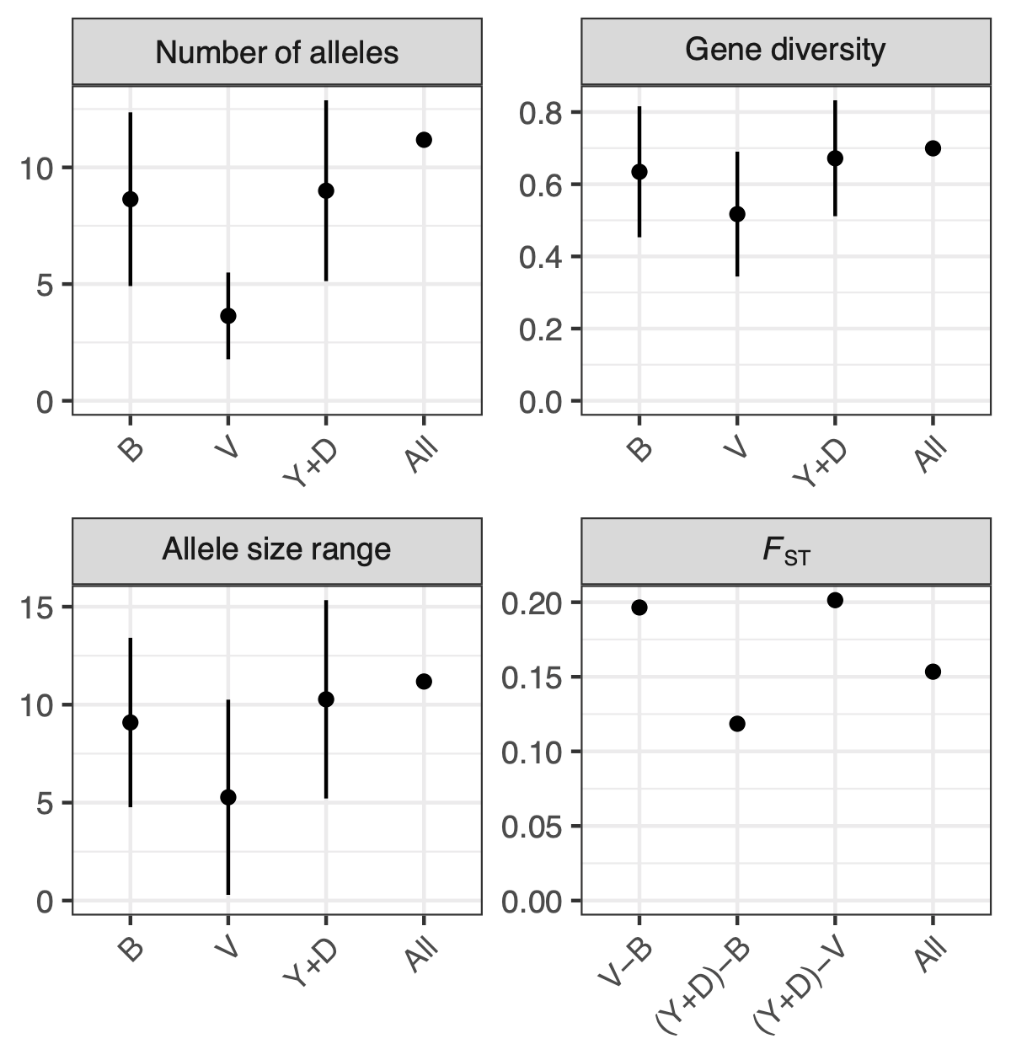


**S6 Fig.** Observed summary statistics. B, Bonin; V, Volcano; Y + D, Yaeyama + Daito groups.
